# LuNER: Multiplexed SARS-CoV-2 detection in clinical swab and wastewater samples

**DOI:** 10.1371/journal.pone.0258263

**Published:** 2021-11-10

**Authors:** Elizabeth C. Stahl, Allan R. Gopez, Connor A. Tsuchida, Vinson B. Fan, Erica A. Moehle, Lea B. Witkowsky, Jennifer R. Hamilton, Enrique Lin-Shiao, Matthew McElroy, Shana L. McDevitt, Alison Ciling, C. Kimberly Tsui, Kathleen Pestal, Holly K. Gildea, Amanda Keller, Iman A. Sylvain, Clara Williams, Ariana Hirsh, Alexander J. Ehrenberg, Rose Kantor, Matthew Metzger, Kara L. Nelson, Fyodor D. Urnov, Bradley R. Ringeisen, Petros Giannikopoulos, Jennifer A. Doudna, IGI Testing Consortium

**Affiliations:** 1 University of California, Berkeley, Berkeley, CA, United States of America; 2 Innovative Genomics Institute, University of California Berkeley, Berkeley, CA, United States of America; 3 Department of Civil and Environmental Engineering, University of California, Berkeley, CA, United States of America; 4 Howard Hughes Medical Institute, University of California, Berkeley, CA, United States of America; Universite du Quebec a Montreal, CANADA

## Abstract

Clinical and surveillance testing for the SARS-CoV-2 virus relies overwhelmingly on RT-qPCR-based diagnostics, yet several popular assays require 2–3 separate reactions or rely on detection of a single viral target, which adds significant time, cost, and risk of false-negative results. Furthermore, multiplexed RT-qPCR tests that detect at least two SARS-CoV-2 genes in a single reaction are typically not affordable for large scale clinical surveillance or adaptable to multiple PCR machines and plate layouts. We developed a RT-qPCR assay using the Luna Probe Universal One-Step RT-qPCR master mix with publicly available primers and probes to detect SARS-CoV-2 N gene, E gene, and human RNase P (LuNER) to address these shortcomings and meet the testing demands of a university campus and the local community. This cost-effective test is compatible with BioRad or Applied Biosystems qPCR machines, in 96 and 384-well formats, with or without sample pooling, and has a detection sensitivity suitable for both clinical reporting and wastewater surveillance efforts.

## Introduction

Diagnostic testing remains a vital component of the public health response to the global pandemic caused by the SARS-CoV-2 [1–7]. Quantitative reverse transcription polymerase chain reaction (RT-qPCR) of RNA extracted from nasal swabs is the most common diagnostic testing method to detect SARS-CoV-2 [8, 9]. Current tests rely on the detection of genomic or sub-genomic viral RNA corresponding to the envelope (E) gene, the nucleocapsid (N) gene, the spike (S) gene, and/or the *ORF1ab* locus [10–12], as well as suitable controls. However, variability in test format as well as reagent availability, quality, and cost continue to hamper widespread use for both symptomatic and asymptomatic testing. One commercially available test entered the scene early, allowing clinical laboratories to rapidly detect multiple viral genes and control RNA in a single reaction; however, this test has reported risks of yielding inaccurate results [13] and suffers from supply shortages and high cost. Another less-expensive test detects only a single viral locus, which can lead to false-negative results [14, 15] and several tests require 2–3 separate PCR reactions to yield clinical results [11, 16, 17]. To address these shortcomings, we developed a new multiplexed assay with optimized viral and control RNA detection using inexpensive, widely available reagents.

An ideal test would offer affordable and robust detection of multiple SARS-CoV-2 genes and a sample extraction control using a single-well multiplexed format. We began our experiments with the Saliva Direct assay [15], which utilizes the CDC’s published PCR primers and probes to detect the SARS-CoV-2 N-gene (N1) and human Ribonuclease P (RNase P) in a 96-well format. We then adapted the test to work in a 384-well format and to accurately detect viral RNA from nasal-swab samples. Finally, we added a third target, SARS-CoV-2 E gene [12], and demonstrated that N gene, E gene, and RNase P could be multiplexed with greater than 95% clinical concordance. This high-throughput and affordable assay provides sensitive detection of SARS-CoV-2 in both clinical samples, extracted individually or in pools of four, and wastewater samples for community surveillance. This approach will be widely useful for monitoring SARS-CoV-2 in the human population and in the environment as the virus becomes endemic around the globe.

## Materials and methods

### Acquisition and processing of clinical samples

The Innovative Genomics Institute Clinical Laboratory is a high-complexity, CLIA-certified laboratory, licensed to operate in the State of California through the California Department of Public Health. We confirm all relevant ethical guidelines have been followed. Descriptive statistics for patient samples used in this manuscript come from de-identified datasets in accordance with human subjects protections, as approved by UC Berkeley’s Committee for Protection of Human Subjects (CPHS). While Institutional Review Board (IRB) approval is required for any research using human subjects, clinical laboratory activities exclusively supporting CLIA-certified clinical operations do not. These activities are governed by CMS and HIPAA legislation. In order to publish our data in this manuscript as results of developing a testing workstream, we sought IRB approval through UC Berkeley’s CPHS. The UC Berkeley Committee for Protection of Human Subjects determined that all the analyses presented in this manuscript do not qualify as human subjects research as the data sets were de-identified to those analyzing them for these results (IRB submission # 2020-04-13177).

### Optimization of multiplexed RT-qPCR assay

Experiments were performed by adding 5μL of positive control plasmid DNA encoding N-gene (2019-nCoV_N_Positive Control, IDT, Coralville, Iowa), RNase P (Hs_RPP30 Positive Control, IDT), E-gene (2019-nCoV_E Positive Control, IDT), and RdRp (2019-nCoV_RdRp (ORF1ab) Positive Control, IDT)) at 200,000 copies/mL directly into 15μL (full reactions, 20μL final volume) or 7.5μL (half reactions, 12.5μL final volume) of master mix in 96 or 384-well plates (ThermoFisher, Waltham, MA).

The master mix was composed of Luna Probe Universal One-Step RT-qPCR (New England Biolabs, Ipswich, MA E3006, E3007, or M3019) and combinations of N1 primers (nCOV_N1 Forward Primer and nCOV_N1 Reverse Primer, IDT), N1 (FAM) probe (nCOV_N1 Probe, IDT), E-Sarbeco primers (E_Sarbeco_F1 Forward Primer and E_Sarbeco_R2 Reverse Primer, IDT), E-Sarbeco (FAM or SUN) probe (E_Sarbeco_P1 Probe, IDT), RdRp primers (RdRP_SARSr_F2 Forward Primer and RdRP_SARSr_R1 Reverse Primer, IDT), RdRp (FAM or SUN) P2 Probe (RdRP_SARSr_P2 Probe, IDT), RNase P primers (RNase P Forward Primer and RNase P Reverse Primer, IDT), and RNase P (ATTO 647) Probe (IDT) diluted in nuclease-free water. The final formulation of LuNER consists of the following: N1 primers and probe (FAM with ZEN/Iowa Black FQ double quenched probe), E-Sarbeco primers and probe (SUN with ZEN/Iowa Black FQ double quenched probe), and RNase P primers and probe (ATTO647 with TAO/Iowa Black RQ double quenched probe), as listed in Table 1.

**Table 1:** Primer and probes tested for the development of the LuNER assay.

	Sequence (5’→3’)	Final Conc. (nM)^a^	Citation
N1 Forward Primer	GACCCCAAAATCAGCGAAAT	400	[11, 15]
N1 Reverse Primer	TCTGGTTACTGCCAGTTGAATCTG	400	[11, 15]
N1 Probe	FAM-ACCCCGCATTACGTTTGGTGGACC-ZEN/IABkFQ	200	[11, 15]
RNase P Forward Primer	AGATTTGGACCTGCGAGCG	150	[11, 15]
RNase P Reverse Primer	GAGCGGCTGTCTCCACAAGT	150	[11, 15]
RNase P Probe	ATTO647-TTCTGACCTGAAGGCTCTGCGCG-TAO/IABkRQ	200	[11, 15]
RdRp Forward Primer	GTGAR ^b^ ATGGTCATGTGTGGCGG	800	[12]
RdRp Reverse Primer	CARATGTTAAAS ^c^ ACACTATTAGCATA	600	[12]
RdRp P2 Probe	SUN-CAGGTGGAACCTCATCAGGAGATGC-ZEN/IABkFQ	100	[12]
E-Sarbeco Forward Primer	ACAGGTACGTTAATAGTTAATAGCGT	400	[12]
E-Sarbeco Reverse Primer	ATATTGCAGCAGTACGCACACA	400	[12]
E-Sarbeco Probe	SUN-ACACTAGCCATCCTTACTGCGCTTCG-ZEN/IABkFQ	200	[12]

^a^ Concentrations are given in nanomole per liter (nM) based on the final reaction mix from 10 μM primer stock solution into 12.5 μL total reaction

^b^ R is G/A

^c^ S is G/C.

The following thermal cycling conditions were used on the CFx96 (Bio-Rad, Hercules CA), QuantStudio-3, and QuantStudio-6 (Applied Biosystems, Foster City, CA): 10 minutes at 52°C, 2 minutes at 95°C, 45 cycles with 10 seconds at 95°C and 30 seconds at 55°C. Data was collected for FAM, Cy5 or ATTO 647 (custom dye), and VIC or SUN. Data was analyzed using the Design and Analysis software (ThermoFisher). Standard thresholds were applied for analysis, N-gene (11,000), E-gene (6,000), RNase P (10,000), across all validation experiments. Ct values were exported into Excel version 16.42 (Microsoft Office, Redmond WA) and plotted with Prism 8.4.3 (Graphpad, San Diego, CA) as the average value ± standard deviation. When comparing Ct values between two groups, a two-tailed Student’s t-test was used. Data were considered statistically significant when p ≤ 0.05. To calculate PCR efficiency, the amount of positive control plasmid (copies/reaction, log scale) was plotted against the average Ct value in Excel to calculate the slope of the linear trend line. The following Eq (1) was used to measure primer efficiency as a percentage, where an efficiency between 90–110% is ideal.


$$Efficiency\;(\%)={(10}^{\frac{-1}{Slope}}-1)\;x\;100$$ (1)

### Sample and control logic and resulting

The LuNER assay was developed, and its performance characteristics were determined in accordance with Clinical Laboratory Improvement Amendments (CLIA), specifically 42 CFR 493.1253. It has been approved for clinical use by the IGI clinical laboratory director and should not be regarded as investigational or for research purposes. This assay has not yet been cleared or approved by the US Food and Drug Administration (FDA), since FDA does not require that this assay go through premarket FDA review, including assays related to COVID-19 [18]. Sample and control logic and resulting are presented in Table 2.

**Table 2:** LuNER assay resulting logic.

Sample Results	Internal Extraction Control	Viral Targets
**Inconclusive**	RNase P Ct < 35	Either E-gene **or** N-gene Ct < 37
		Queued for retest
**Invalid**	RNase P Ct ≥ 35	Both E-gene **and** N-gene Ct ≥ 37
		Queued for retest
**Negative**	RNase P Ct < 35	Both E-gene **and** N-gene Ct ≥ 37
**Positive**	Ignored ^a^	Both E-gene **and** N-gene Ct < 37
		Either E-gene **or** N-gene consistently detected at Ct < 37 (when retested from Inconclusive)
**Plate Controls**		**Valid when**
**Extraction Negative (NC)**		RNase P Ct ≥ 35, both E-gene **and** N-gene Ct ≥ 37
**Extraction Human RNA (HC)**		RNase P Ct < 35, both E-gene **and** N-gene Ct ≥ 37
**RT-qPCR Negative**		RNase P Ct ≥ 35, both E-gene **and** N-gene Ct ≥ 37
**RT-qPCR positive**		RNase P Ct ≥ 35, both E-gene **and** N-gene Ct < 37

^a^ RNase P target may be outcompeted by SARS-CoV-2 genes when present at high abundance. Therefore, amplification of RNase P can be ignored in accordance with the CDC assay [11].

Samples within a pool are flagged to be re-tested individually when N-gene or E-gene amplify at Ct values < 37 (RNase P is ignored) in the pool; and also, when RNase P fails to amplify (Ct ≥ 35) in the pool. Otherwise, if RNase P has a Ct value < 35 and both N-gene and E-gene fail to amplify or have Ct values ≥ 37, all four samples within the pool are resulted as negative. Ct values may be returned as “undetermined” if there is no amplification (treated the same as Ct ≥ 37 for N-gene and E-gene or ≥ 35 for RNase P).

An individual sample is called negative when N gene and E gene have Ct values ≥ 37 and RNase P has a Ct value < 35. An individual sample is called invalid when N gene and E gene have Ct values ≥ 37 and RNase P has Ct ≥ 35. Individual samples that result as invalid are then retested from RNA extraction. If the sample fails to amplify RNase P a second time, a final result “Specimen Insufficient” is given and a new sample is typically ordered. An individual sample is called inconclusive when N gene or E gene have Ct values < 37 and RNase P < 35. Inconclusive samples are then retested from RNA extraction; and will be resulted as positive if the same viral target has Ct values < 37, or negative if N gene and E gene have Ct values ≥ 37 and RNase P has a Ct value < 35. For samples where one viral target has Ct < 37 in the initial test, and the other viral target has Ct < 37 in the retest, we consider the viral load to be below our LoD and therefore negative, as we are unable to obtain a consistent result. An individual sample is called positive when both N gene and E gene have Ct values < 37 or when one of the viral targets consistently amplifies at Ct values < 37 (when retesting from Inconclusive) and RNase P is ignored.

Two controls undergo the full process of RNA extraction per 96-well plate. The “NC” control (50:50 2xRNA Shield:1xPBS) is valid when N gene and E gene have Ct values ≥ 37 and RNase P has Ct ≥ 35, controlling for background amplification or contamination. The “HC” control (50:50 2xRNA Shield:1xPBS with 400 nanograms of Universal Human Reference RNA (Thermo Fisher)) is valid when N gene and E gene have Ct values ≥ 37 and RNase P has a Ct value < 35, controlling for non-specific amplification, contamination, and retention of genomic material.

Prior to RT-qPCR, four wells in the 384-well plate receive nuclease-free water as “qPCR Negative Controls,” which are valid when N gene and E gene have Ct values ≥ 37 and RNase P has Ct ≥ 35. Additionally, four wells receive positive RNA control SARS-CoV-2 synthetic control 2 (MN908947.3, Wuhan-Hu-1, Twist Bioscience) diluted in nuclease-free water to 100 copies/μL for 500 copies per reaction and are valid when N gene and E gene have Ct values < 37 and RNase P has a Ct value ≥ 35.

### Sample pooling and RNA extraction

Oropharyngeal (OP, Improve Medical, China) swabs were used to collect OP/mid-turbinate samples from individuals at walk-up testing centers in Berkeley, CA. Swabs were transported to the IGI Clinical Laboratory in 2mL of 2x DNA/RNA Shield (Zymo Research, Irvine CA), diluted to 1x with sterile phosphate buffered saline, pH 7.4 (Gibco, Gaithersburg, MD). Samples were kept at 4°C until 450μL (single extractions) or 112.5μL (fourplex pooled extractions) was aliquoted into 96-well deep-well plates (Axygen, San Francisco CA) with the Microlab STARlet liquid-handling robot (Hamilton, Reno NV). RNA extraction was performed on the Vantage liquid handling system (Hamilton) with the MagMax Viral/Pathogen Nucleic Acid Isolation Kit (Applied Biosystems), according to the manufacturer’s instructions, with the exception of omitting MS2 spike-in for the LuNER assay. Final RNA elution volume was 22μL. RNA was stored at -80°C or immediately arrayed into 384-well master mix plates on the Vantage liquid-handling system (5μL of sample into 7.5μL master mix) for RT-qPCR.

### Limit of detection

Heat-inactivated virus was generated in the BSL3 laboratory at the University of California, Berkeley. Briefly, SARS-CoV-2 was cultured in Vero E6 cells and titers were determined using cytopathic effect (CPE) assays. Viral samples were incubated at 37°C for 30 minutes in the presence of 0.5 mg/mL proteinase K, followed by incubation at 75°C for 30 minutes to inactivate. The starting titer was 158,000 TCID_50_/mL. To determine the limit of detection, twelve clinically reported negative samples (OP/mid-turbinate swabs in 2mL of 1x RNA/DNA shield) were pooled to generate a negative sample matrix for spike-in experiments. 45μL of 10x heat-inactivated virus was added to 405μL of sample matrix to generate 450μL of material for RNA extraction using the methods described above at final concentrations of approximately 50–0.01 TCID_50_/mL in triplicate. For pooling experiments, 112.5uL of the contrived positive samples were mixed with 337.5uL of negative clinical matrix. Following RNA extraction, the samples were arrayed into the LuNER master mix as described above and RT-qPCR was performed on the QuantStudio-6. Data was analyzed using the Design and Analysis software (ThermoFisher). Ct values were exported into Excel version 16.42 (Microsoft Office, Redmond WA) and plotted with Prism 8.4.3 (Graphpad, San Diego, CA) as the average value ± standard deviation.

### Reproducibility

Sixteen clinically reported negative samples (OP/mid-turbinate swabs in 2mL of 1x RNA/DNA shield) were pooled to generate a negative sample matrix for spike-in experiments. 45μL of 10x heat-inactivated virus was added to 405μL of sample matrix to generate 450μL of contrived sample. Then 112.5μL of the contrived positive samples were mixed with 337.5μL of negative clinical matrix to simulate fourplex sample pooling. RNA extraction was performed using the methods described above at final concentrations of 2.56, 1.28, and 0.64 TCID_50_/mL with twenty-replicates each. Following RNA extraction, the samples were arrayed into the LuNER master mix as described above and RT-qPCR was performed on the QuantStudio-6. Data was analyzed using the Design and Analysis software (ThermoFisher). Ct values were exported into Excel version 16.42 (Microsoft Office, Redmond WA) and plotted with Prism 8.4.3 (Graphpad, San Diego, CA) as the average value ± standard deviation.

### Clinical concordance

240 OP/mid-turbinate samples with clinically reported results based on the TaqPath RT-PCR COVID-19 kit (ThermoFisher) were arrayed from the original tubes into a 96-well deep-well plate on the Microlab STARlet. Sample tubes were loaded into the STARlet with a confirmed positive sample spaced every four tubes, leading to 30 fourplex pools (each containing one positive sample) followed by 30 fourplex pools (each containing only negative samples). RNA was extracted from the pooled samples, as described above, followed by RT-qPCR with LuNER. Pooled sample results were analyzed and all samples that showed amplification of either N-gene or E-gene were retested from RNA extraction as singlets.

### Wastewater surveillance

Twenty-four-hour composite influent samples from nine San Francisco Bay Area sampling sites were obtained. These samples were extracted as previously reported [19] and detailed in depth at dx.doi.org/10.17504/protocols.io.bpdfmi3n. Briefly, samples were treated with sodium chloride (NaCl) to a final concentration of 4 M, ethylenediamine tetraacetic acid (EDTA) to 1 mM, and pH 7.2 tris(hydroxymethyl)aminomethane to 10 mM. Samples were then incubated at 70°C for 45 minutes and passed through a 5 μM DuraPore PVDF filter (Millipore Sigma), followed by a syringe filter. Ethanol was added to a final concentration of 35%. This mixture was pulled through Zymo-IIIP columns (Zymo Research) by a vacuum manifold. Columns were washed sequentially with 25 mL 4S-WB1 (1.5 M NaCl, 20% ethanol) and 50 mL 4S-WB2 (100 mM NaCl and 80% ethanol). After washing, columns were centrifuged at 10000 x g for 2 minutes, and RNA was eluted with 200 μL prewarmed ZymoPURE elution buffer (Zymo Research). Eluted RNA was stored at 4°C for use in same-day qPCR assays.

The COVID-WEB pop-up laboratory currently uses the CDC SARS-CoV-2-N1 primer/probe set with the NEB Luna Universal One-Step RT-qPCR Kit (New England BioLabs E3005) per manufacturer’s instructions. Reactions are cycled at 25°C for 2 minutes, 50°C for 15 minutes, 95°C for 2 minutes, and 45 cycles of [95°C for 3 seconds followed by 55°C for 30 seconds]. Three technical replicate RT-qPCR wells are analyzed for each sample and standard. This method was used on the QuantStudio-3 (Applied Biosystems) to compare to LuNER.

## Results and discussion

### Development of the LuNER assay

TaqMan-based RT-qPCR relies on the amplification of a reverse-transcribed product quantified in real-time by release of a fluorescent dye from a probe. By labeling different probes with spectrally distinct fluorescent dyes, such as FAM, VIC/HEX/SUN, and Cy5/ATTO647, PCR reactions can be multiplexed to detect three different targets in a single reaction (Table 1).

**Fig 1:**
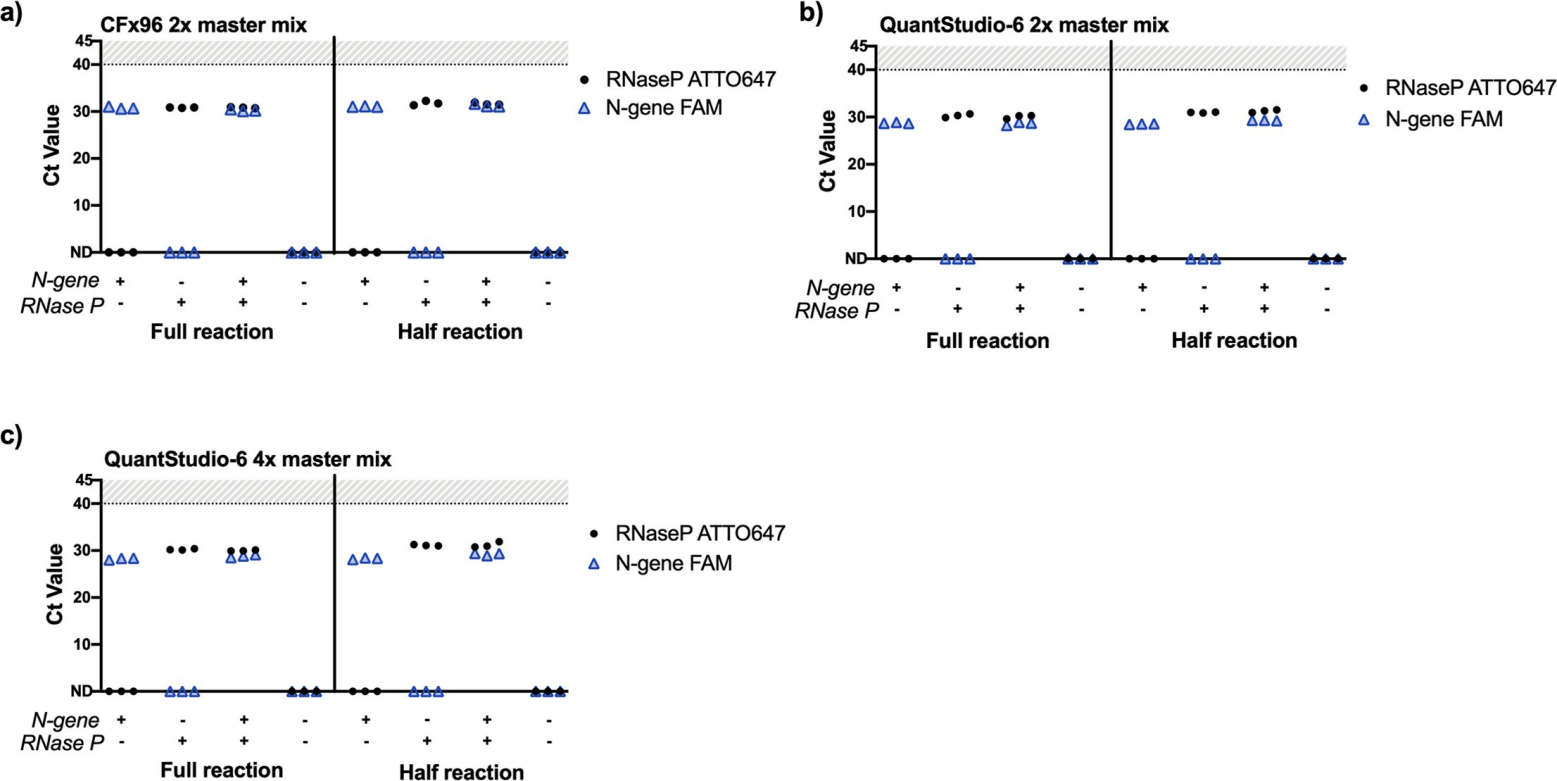
Implementation and adaptation of the Saliva Direct RT-qPCR assay to detect SARS-CoV-2 N1 and human RNase P with the QuantStudio-6. a) Full and half reaction volumes on the CFx96 with 2x master mix, b) Full and half reaction volumes on the QuantStudio-6 with 2x master mix, c) Full and half reaction volumes on the QuantStudio-6 with 4x master mix. Samples which failed to amplify are denoted as “not detected” (ND). Plasmid DNA was added directly into the master mix at 200,000 copies/mL (2-4x Saliva Direct limit of detection) in triplicate. Full reactions (15μL master mix with 5μL sample input), half reactions (7.5μL master mix with 5μL sample input).

To establish published duplex reactions [15] (i.e. two distinct PCR targets per well) in our facility, RT-qPCR was first performed on contrived samples for N1 and RNase P using the Bio-Rad CFx96 with the recommended full (20μL) reaction volume. Amplification of N1 and RNase P was observed in duplexed reactions using either the full or half-reaction volumes (Fig 1A). Half-reaction volumes were tested in order to save reagents and also facilitate a transition to 384-well plates. The duplexed reactions were then successfully adapted to the Applied Biosystems QuantStudio-6 using a 384-well plate format after custom dye calibration to detect ATTO 647 (Fig 1B and 1C), with both the standard 2x and a newly developed 4x Luna Probe One-Step RT-qPCR master mix from New England BioLabs. This more concentrated 4x master mix contains a passive reference dye that is compatible across many instruments as well as dUTP to prevent carryover of contaminants and allows for larger sample input and multiplexing.

There was no change in the average Ct values between the 2x and 4x master mix formulations on the QuantStudio-6; however, Ct values for N1 increased by an average of 0.5 and RNase P by 1 in the duplexed, half-reaction volume compared to full reaction volume. Taken together, these data establish that duplexed reactions to detect N1 and RNase P can be adapted from 96 to 384-plate format and alternate instrumentation with minimal loss of sensitivity, allowing for four-times greater sample testing in one RT-qPCR run.

**Fig 2:**
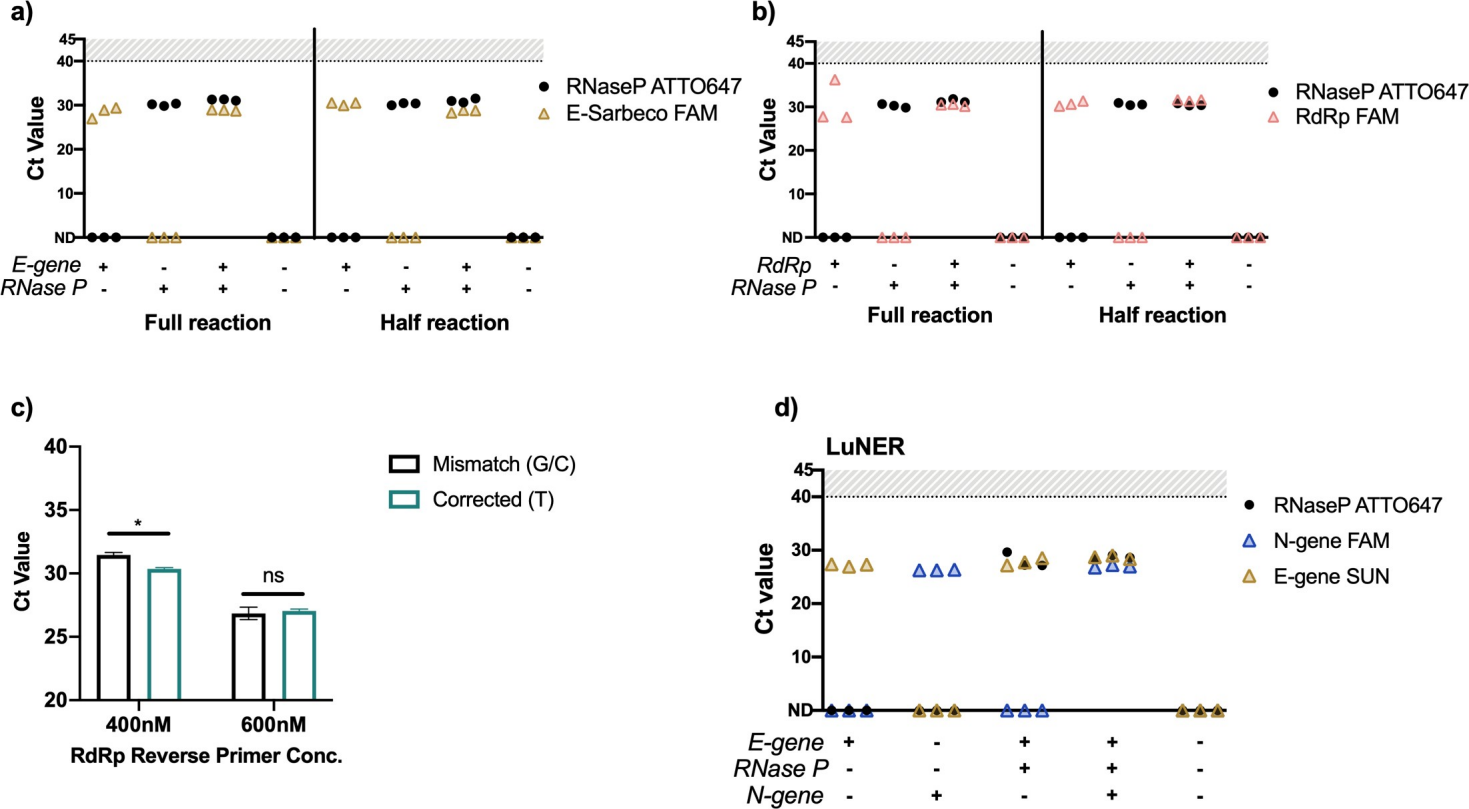
Evaluating E-Sarbeco and RdRp-SARSr primers and probes with RNase P on the QuantStudio-6. a) Full and half-sized reactions of E-Sarbeco on the QuantStudio-6 with 4x master mix, b) Full and half-sized reactions of RdRp on the QuantStudio-6 with 4x master mix. Plasmid DNA was added directly into the master mix at 200,000 copies/mL (2-4x Saliva Direct limit of detection) in triplicate. Full reactions (15μL master mix with 5μL sample input), half reactions (7.5μL master mix with 5μL sample input). c) Evaluation of corrected RdRp reverse primers at two different concentrations on pooled RNA eluted from positive clinical swab samples in triplicate. p<0.001 (*) between mismatch and corrected at 400nM and between both concentrations of primers. Not significant (ns). d) E-Sarbeco primers and probes multiplexed with N1 and RNase P in a single half-sized reaction on the QuantStudio-6 with 4x master mix was selected as the final formulation for LuNER.

Using a single target to detect the presence of SARS-CoV-2 in a specimen may increase the risk for false-negative results [14]. One way to improve this is by integrating a second viral target into the assay, such as the N2 nucleocapsid locus [20]; however, published data show that detection of N2 by RT-qPCR is less sensitive than N1 [16], increased likelihood of N2 primers to form dimers [21], and homology to SARS-CoV-1 in the N2 forward primer [22]. To diversify the viral RNA regions sampled in our assay, both E gene and the RNA-dependent RNA polymerase (RdRp) locus of *ORF1ab* were evaluated [12]. RdRp-SARSr and E-Sarbeco (Charité) were successfully combined with RNase P in duplexed, half-reactions (Fig 2A and 2B).

The RdRp primers are recommended to be used at high concentrations of 600-800nM [12] and were found to be less sensitive than other commonly used SARS-CoV-2 reagents, potentially due to a mismatch between the reverse primer and viral genome [16]. In order to control for the mismatch, we designed custom primers where S (G/C) at position 12 was corrected to T. Correcting the mismatch improved the average Ct value by 1.1 (from Ct = 31.4 to 30.3) when the reverse primer was tested at a final concentration of 400nM (p<0.01, Fig 2C). Interestingly, at the 600nM concentration there was no difference in Ct values between the mismatch and corrected primers (Ct = 26.8 to 27.0), suggesting the mismatch is not the primary cause of reduced sensitivity. The necessity of using high primer concentrations for the RdRp target makes it impossible to multiplex with N1 and RNase P using the standard 2x master mix. Multiplexing was achieved using the 4x master mix by increasing the input concentrations of primers and probes. These data suggest multiplexing with existing RdRp primers and probes is challenging but can be achieved if reagents are amenable to smaller input volumes.

The E-Sarbeco primers and probes were easily multiplexed in PCR reactions using both the 2x and 4x master mix formulations at the standard input concentration of 10μM (Fig 2D) for a final primer concentration of 400nM, demonstrating superior performance over RdRp. Therefore, we have selected the 4x Luna master mix to detect N-gene, E-gene, and RNase P (NER) as the final formulation of our multiplexed assay, hereafter referred to as LuNER. Validation studies using the LuNER assay were performed to define the limit of detection, demonstrate reproducibility, and determine clinical concordance.

### LuNER validation

Validation of the LuNER assay was originally performed using contrived or clinical nasal-swab samples extracted and tested individually using a Ct cutoff of 40 [23]. When performed on individual samples, the LuNER assay was found to have a sensitive limit of detection (LoD, 0.5 TCID_50_/mL) and high clinical concordance with another commercially available test for SARS-CoV-2 detection [23] (S1 and S2 Figs in S1 File, S1 Table in S2 File).

Here we report additional validation studies that were performed on clinical samples pooled in groups of four prior to RNA extraction and target detection. To continuously iterate on the assay design, we implemented an intermediate “inconclusive” result for samples that are originally found with only one of two viral genes, requiring a second test before reporting the sample as positive. Additionally, we found that a lower Ct cutoff of 37 had minimal impact on assay performance for positive detection, but markedly reduced the number of inconclusive samples that resulted negative upon retesting (S2 and S3 Table in S2 File).

To measure the LoD of LuNER in sample pools of four, heat-inactivated SARS-CoV-2 was introduced into negative matrix generated from one clinically reported negative sample. A quarter of this contrived positive sample was combined with equal volumes of three additional negative samples to mimic the pooling protocol. The final volume of the pooled sample is equal to the single sample input for RNA extraction; therefore, the amount of viral material is diluted four-fold and the LoD is expected to be four times higher than the single swab assay. RNA extraction was performed with the MagMax Viral/Pathogen Nucleic Acid Isolation Kit using the Hamilton Vantage liquid-handling system, as described previously [24].

**Fig 3:**
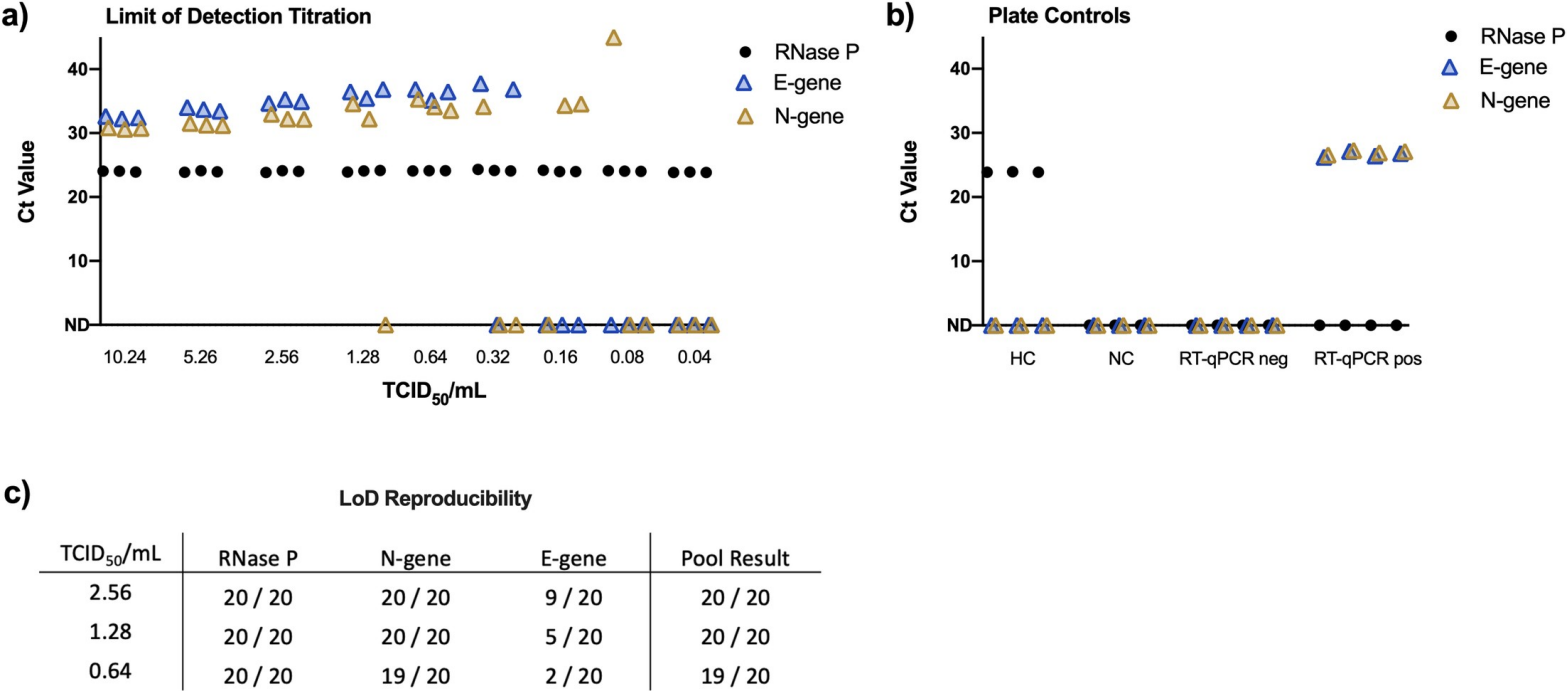
Limit of detection and reproducibility. a) The limit of detection was defined by extracting RNA from heat-inactivated virus at concentrations ranging from 10.24 to 0.01 TCID_50_/mL and performing RT-qPCR with the LuNER reagents. b) Representative plate controls for the LuNER assay are shown from the LoD titration experiment, including negative buffer-only control (NC), human RNA control (HC), RT-qPCR negative (blank), and RT-qPCR positive controls (synthetic SARS-CoV-2 RNA genome). c) An independent experiment showing reproducibility of the LoD at 0.64, 1.28, and 2.56 TCID_50_/mL.

Following RNA extraction, pooled samples were analyzed with the LuNER RT-qPCR assay. Since detection of only a single viral gene is sufficient to identify a pool for individual sample testing, so we set out to determine the lowest amount of virus required to flag a pool. Either N-gene or E-gene were detectable in triplicates at increasing Ct-values from 10.24 to 0.64 TCID_50_/mL (Fig 3A), along with valid extraction and RT-qPCR controls (Fig 3B). At 0.32 TCID_50_/mL, one of the three replicates was negative for both E-gene and N-gene, therefore we tested 20 replicates at 0.64, 1.28, and 2.56 TCID_50_/mL to assess reproducibility (Fig 3C). At 0.64 TCID_50_/mL, only 19/20 samples amplified either N-gene or E-gene. At both 1.28 and 2.56 TCID_50_/mL, 20/20 samples amplified either N-gene or E-gene, therefore the lowest concentration to flag a pool for individual sample testing was 1.28 TCID_50_/mL. Both experiments demonstrate that N1 is more sensitive than E-Sarbeco at very low concentrations of virus.

Next, to assess how the LuNER assay performs on clinical samples, we generated 60 pooled wells (240 individual samples), where 30 wells were expected to contain a single positive sample and 30 wells were expected to contain no positive samples. The expected sample status is based on previous results from testing with the TaqPath RT-PCR COVID-19 kit from ThermoFisher. Samples were pooled using the Hamilton STARlet liquid-handling system, as described previously [25] by combining equal volumes from four sample tubes into a single well in a 96-deep well plate. Then the plates underwent RNA extraction and RT-qPCR with LuNER.

**Fig 4:**
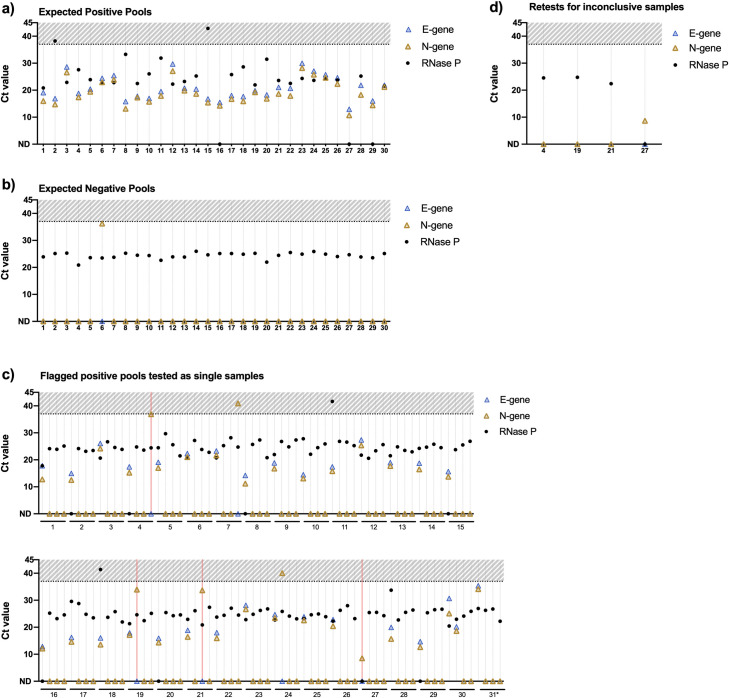
Clinical concordance. a) One hundred and twenty samples were pooled into thirty wells, each expected to contain one positive sample, and tested for SARS-CoV-2 with the LuNER assay, which flagged all thirty wells for testing individually, b) One hundred and twenty independent samples were pooled into thirty wells, each expected to contain zero positive samples, and tested for SARS-CoV-2 with the LuNER assay, which flagged one well for testing individually, c) Individual testing results for 124 samples flagged from pooled wells (pink lines denote inconclusive results, asterisk denotes expected negative pool 6), d) Four samples that amplified only N-gene were retested and resulted accordingly. Overall clinical concordance with the previous sample result was 99.6%, with one additional positive sample identified with LuNER.

All 30 expected positive pools resulted with both N-gene and E-gene (Fig 4A), while 29/30 expected negative pools resulted without N-gene or E-gene (Fig 4B). Samples from these 31 “flagged pools” (124 samples) were then re-extracted and tested individually with LuNER.

Each pool is expected to contain a single positive sample; however, four pools were found to contain an additional positive or inconclusive sample and one pool contained one inconclusive sample only (Fig 4C). The additional positive sample identified in pool 30 was determined to be a true positive based on the original result with TaqPath qRT-PCR and was included in this experiment by error.

Samples from pools 4, 19, 21, and 27 only amplified N-gene and were thus marked Inconclusive (pink lines) and were sent through an additional round of extraction and RT-qPCR analysis (Fig 4D). After retesting, the Inconclusive samples in pools 4, 19, and 21 did not amplify either N-gene or E-gene, so the final sample result was negative, concordant with the original sample result. The sample in pool 27 amplified N-gene at a consistently low Ct value (8.5–8.6 individually, 10.7 in the pool), so the final sample result was positive, also concordant with the original sample result. This result may demonstrate PCR inhibition of E-Sarbeco primers/probe when the sample is too concentrated.

Finally, the samples from expected negative pool 6 were tested individually (denoted in Fig 4C as pool 31). Indeed, one of the four samples was positive (N-gene Ct = 34.1, E-gene Ct = 35.4). Since LuNER is more sensitive than the previous assay used to determine the original sample result (S2 Fig in S1 File and S1 Table in S2 File) and this sample has high Ct viral genes, it is likely that we identified an additional positive sample with LuNER that was previously missed. Out of 240 samples tested using our pooling pipeline, the final sample results were identical in 239/240 samples (99.6% concordance), with one previously negative sample now testing positive.

### LuNER for wastewater testing

Investigators at UC Berkeley established the “COVID wastewater epidemiology for the Bay Area (COVID-WEB)” pop-up testing laboratory for SARS-CoV-2 to identify the presence of the virus in human waste [19]. Currently viral load is measured from wastewater by RT-qPCR for the N1 target using a QuantStudio-3 machine and 96-well layout. However, multiplexing the RT-qPCR will allow for collection of additional data from the wastewater specimens.

**Fig 5:**
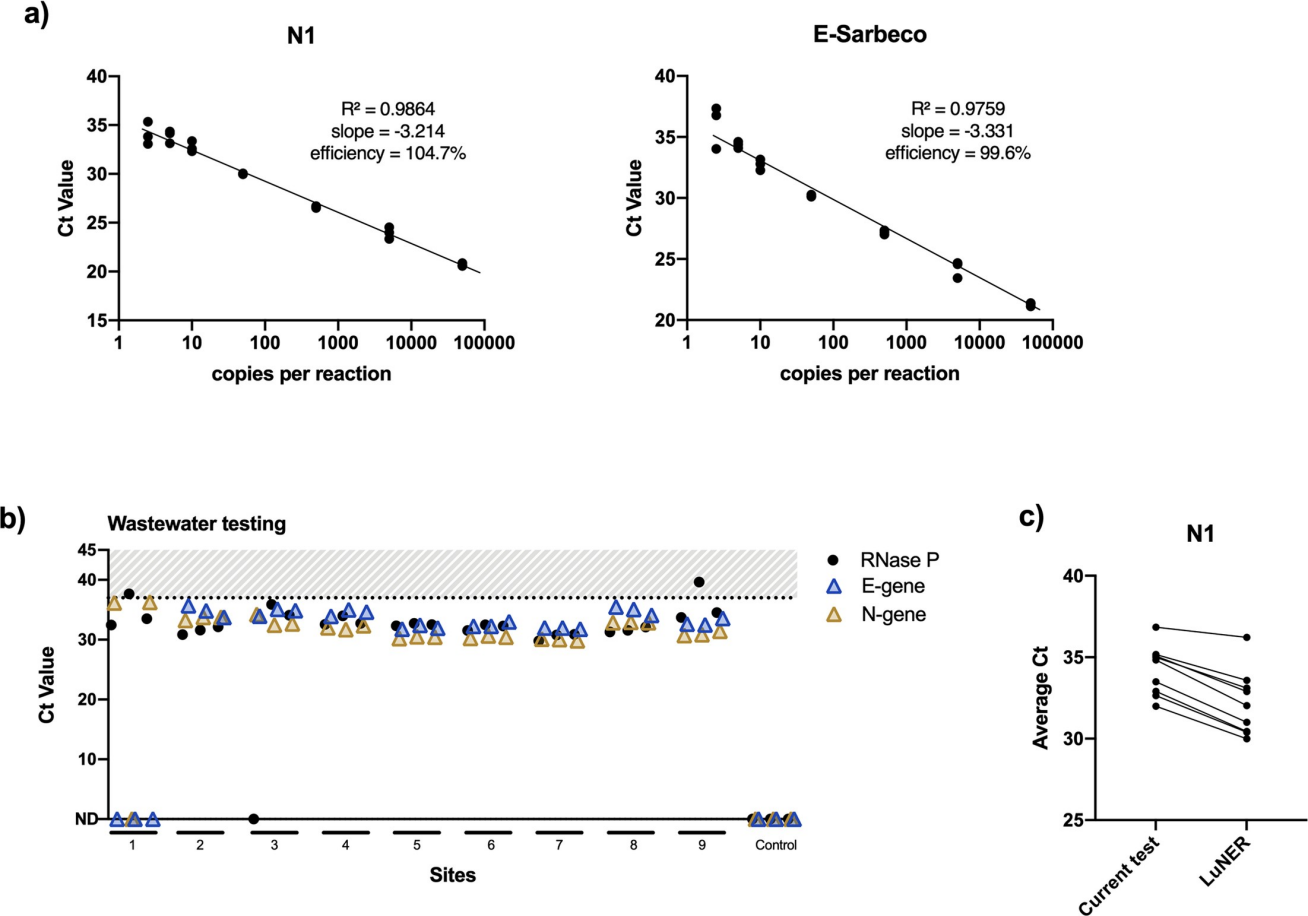
Wastewater testing with LuNER. a) PCR efficiencies of LuNER measured on the QuantStudio-3 using control plasmids ranging from 2.5–50,000 copies per reaction. Slopes between -3.1 and -3.6 giving reaction efficiencies between 90 and 110% are typically acceptable, b) Nine wastewater sites around the San Francisco Bay Area were surveilled for SARS-CoV-2 with LuNER on the QuantStudio-6, c) Comparing Ct Values for N1 between the test currently deployed by the pop-up wastewater lab and LuNER for each of the nine sites.

To test the applicability of LuNER for wastewater surveillance, the assay was deployed in two different formats: 96-well plates on the QuantStudio-3 and 384-well plates on the QuantStudio-6 using the half-reaction volumes. When LuNER was first tested by the pop-up lab on the QuantStudio-3, the PCR efficiencies of N1 and E-Sarbeco were high in the complete master mix (104.7% and 99.6% respectively, Fig 5A).

As the QuantStudio-3 does not have the ability to measure RNase P in Cy5/ATTO647, reactions were then tested on the QuantStudio-6 in the 384-well format with freshly extracted wastewater samples. Wastewater collected from nine different sites was found to contain viral RNA with LuNER, as expected (Fig 5B). Human RNase P could be detected in the wastewater samples with average Cts ranging from 30.5 to 36.0 from site-to-site. Average Ct values for N-gene ranged from 30.0–36.2 and E-gene ranged from 32.0–34.9 with RNA from undiluted wastewater samples.

While clinical samples are not tested in replicate, the wastewater samples tested in triplicate showed some variability. Replicates of site 1 amplified only N-gene and not E-gene, with one replicate losing N-gene amplification, leading to an overall inconclusive sample result, consistent with the current test used by COVID-WEB. Additionally, one replicate from site 3 did not amplify RNase P, and replicates from sites 1 and 9 showed variability in RNase P. Despite the N-gene drop-out in site 1, when paired Ct values were compared between the current test and LuNER, the N-gene Ct values were generally lower with LuNER (Fig 5C). Taken together, these results show that LuNER can be deployed for duplexed RT-qPCR on the QuantStudio-3 or multiplexed RT-qPCR measurement of wastewater samples on the QuantStudio-6.

## Conclusions

The SARS-CoV-2 pandemic has cast an unprecedented level of attention on technical specifications of PCR-based tests as well as their affordability and supply chain dependence. One of the drawbacks of PCR diagnostics is the lengthy turnaround time due to the complexity of molecular biology techniques involved. Performing PCR reactions in a 384-well format allows for a greater number of samples to be processed each day, resulting in shorter turnaround times, and also reduces the cost per sample by lowering reagent volumes and thus supply chain dependence. While other groups have adopted extraction-free methods, 384-well formats, or multiplexed PCR reactions to improve turnaround times, these assays are limited by the fact that they 1) target a single site in the viral genome, 2) require 2–3 separate PCR reactions, or 3) utilize proprietary primer and probe sequences that are expensive and/or not widely available [26].

The LuNER assay addresses these shortcomings by combining affordable, publicly and commercially available SARS-CoV-2 reagents into a multiplexed 384-well reaction. We show that the LuNER assay has improved sensitivity and greater than 95% clinical concordance compared to a similar multiplexed assay. The LuNER assay is estimated to cost $1.26 per reaction, a 12x reduction in cost from other commercially available multiplexed tests and 48x reduction in cost when combined with fourplex pooling. The LuNER assay greatly reduces the number of “invalid” results by implementing an internal human extraction control for further cost and reagent savings.

Importantly, there are limitations to the LuNER assay. The RNase P primers designed by the CDC may amplify both RNA and genomic DNA [27]; thus using a redesigned RNase P reverse primer that specifically detects RNA or incorporating DNase digestion of the sample prior to RT-qPCR may improve the test further. Although the limit of detection was measured with biologically relevant inactivated virus, an estimate of the relative sensitivity in genomic copies can be gained by the comparison to the TaqPath assay (S2 Fig in S1 File), which we previously found to have an LoD of 1 copy per microliter (5 copies per reaction) [24], and additional experiments with contrived samples (Fig 5) where N-gene and E-gene were detected at 2.5 copies per reaction. Finally, pooling samples increases the limit of detection of the assay and thus samples at high Ct values may no longer be detectable. In our validation, we tested sample pools with known individual Ct values for N-gene ranging from 7.16 to 33.72, covering one standard deviation of the average Ct values collected by our laboratory. However, testing of samples with Ct values greater than 35 should be conducted to further assess sensitivity.

Diagnostic testing for SARS-CoV-2 will remain necessary for the foreseeable future. The LuNER assay will improve accessibility for SARS-CoV-2 testing by reducing costs and can be easily adapted to detect new SARS-CoV-2 variants or novel pathogens in the future.

## Supporting information

S1 Graphical abstract (TIF)

S1 File Limit of detection, reproducibility, clinical concordance for samples extracted individually. (PDF)

S2 File Raw data from all experiments in the manuscript. (PDF)
