# Supplementary figures and images for: LuNER: Multiplexed SARS-CoV-2 detection in clinical swab and wastewater samples
Source: PLoS One. 2021 Nov 10;16(11):e0258263. doi: 10.1371/journal.pone.0258263 (PMC8580221; doi:10.1371/journal.pone.0258263)

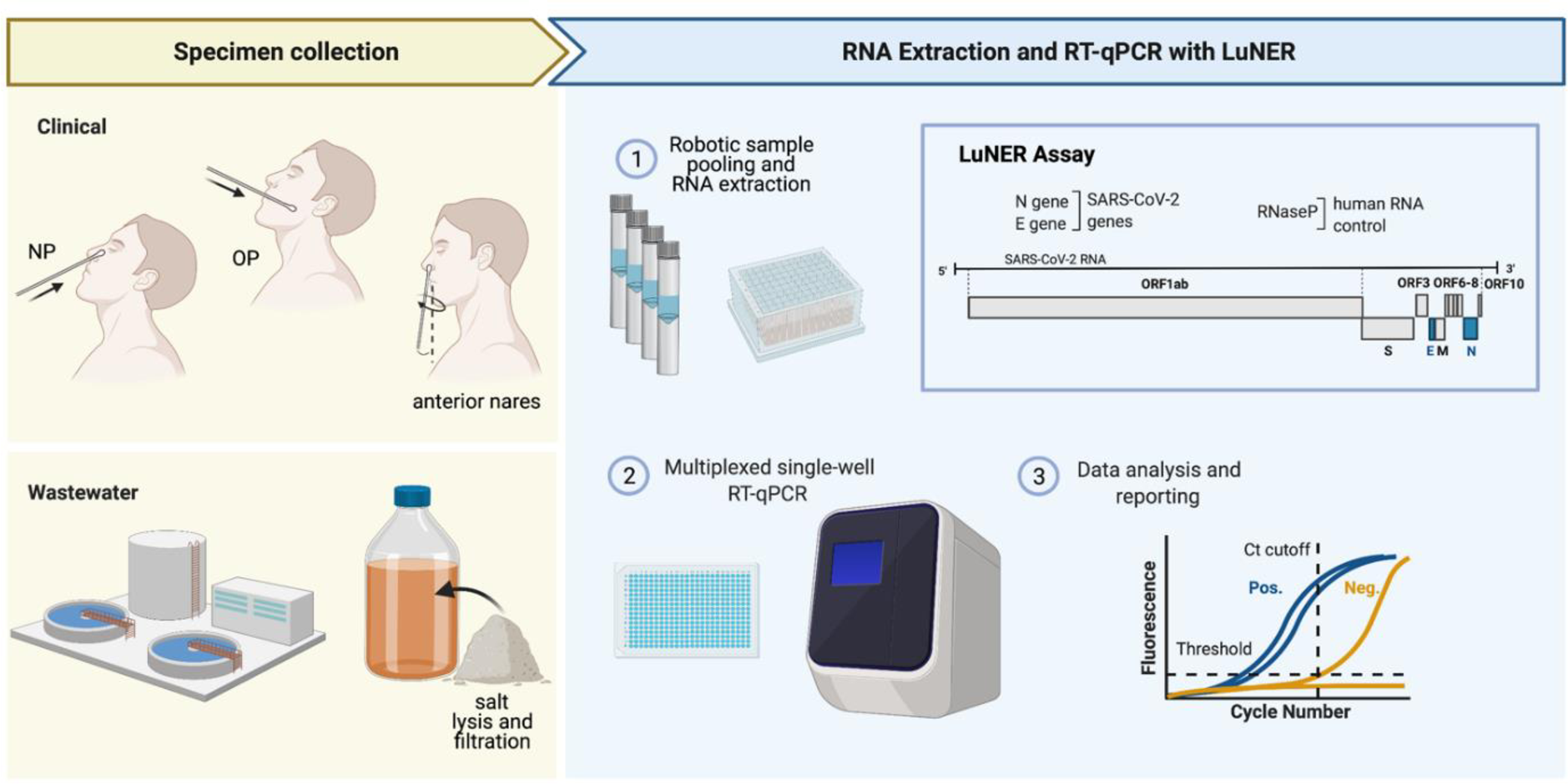

Supplement: S1 Graphical abstract — (TIF) [file pone.0258263.s001.tif]
