# Supplementary material for: LuNER: Multiplexed SARS-CoV-2 detection in clinical swab and wastewater samples
Source: PLoS One. 2021 Nov 10;16(11):e0258263. doi: 10.1371/journal.pone.0258263 (PMC8580221; doi:10.1371/journal.pone.0258263)
Supplement: S1 File — (PDF) [file pone.0258263.s002.pdf]

LuNER: multiplexed SARS-CoV-2 detection in clinical swab and wastewater samples

Elizabeth C. Stahl[1,2], Allan R. Gopez[2], Connor A. Tsuchida[1,2], Vinson B. Fan[1], Erica A. Moehle[1,2], Lea B. Witkowsky[1,2], Jennifer R. Hamilton[1,2], Enrique Lin-Shiao[1,2], Matthew McElroy[2], Shana L. McDevitt[1,2], Alison Ciling[1,2], C. Kimberly Tsui[1], Kathleen Pestal[1], Holly K. Gildea[1], Amanda Keller[2], Iman A. Sylvain[2], Clara Williams[2], Ariana Hirsh[1,2], Alexander J. Ehrenberg[1], Rose Kantor[1], Matthew Metzger[1], IGI Testing Consortium[2^], Kara L. Nelson[1,2], Fyodor D. Urnov[1,2], Bradley R. Ringeisen[1,2], Petros Giannikopoulos[2], Jennifer A. Doudna[1,2,3*]

[1]University of California, Berkeley, Berkeley, CA, USA. [2]Innovative Genomics Institute, University of California Berkeley, Berkeley, CA, USA. [3]Howard Hughes Medical Institute, University of California, Berkeley, CA, USA.

^ Membership of the IGI Testing Consortium is provided in the acknowledgements

*Corresponding author

Email: doudna@berkeley.edu

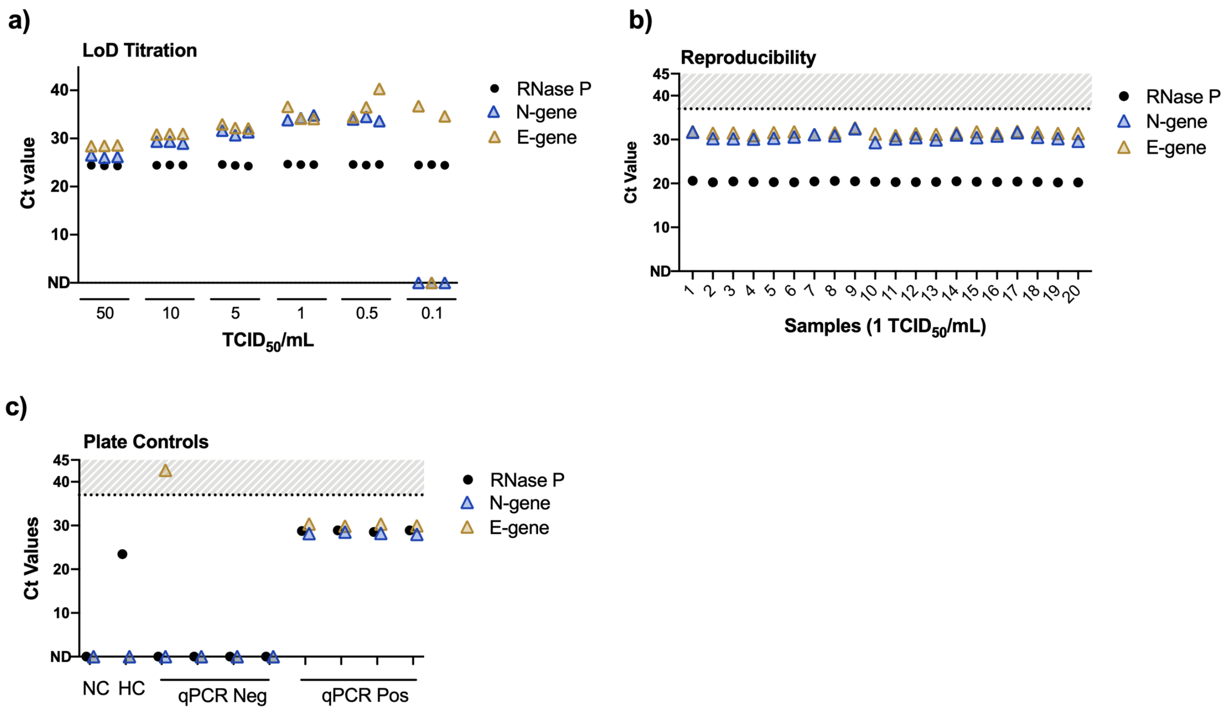

S1 Fig. Limit of detection and reproducibility for samples extracted individually. a) The limit of detection was defined by extracting RNA from heat inactivated virus at concentrations ranging from 50 $TCID_{50}$/mL to 0.1 $TCID_{50}$/mL and performing RT-qPCR with the LuNER reagents. b) Twenty replicates at 1 $TCID_{50}$/mL were prepared by extracting RNA from heat inactivated virus and performing RT-qPCR with the LuNER reagents to test for reproducibility. c) Controls for the LuNER assay are valid, including negative buffer-only control (“NC”), human RNA control (“HC”), qPCR negative, and qPCR positive controls.

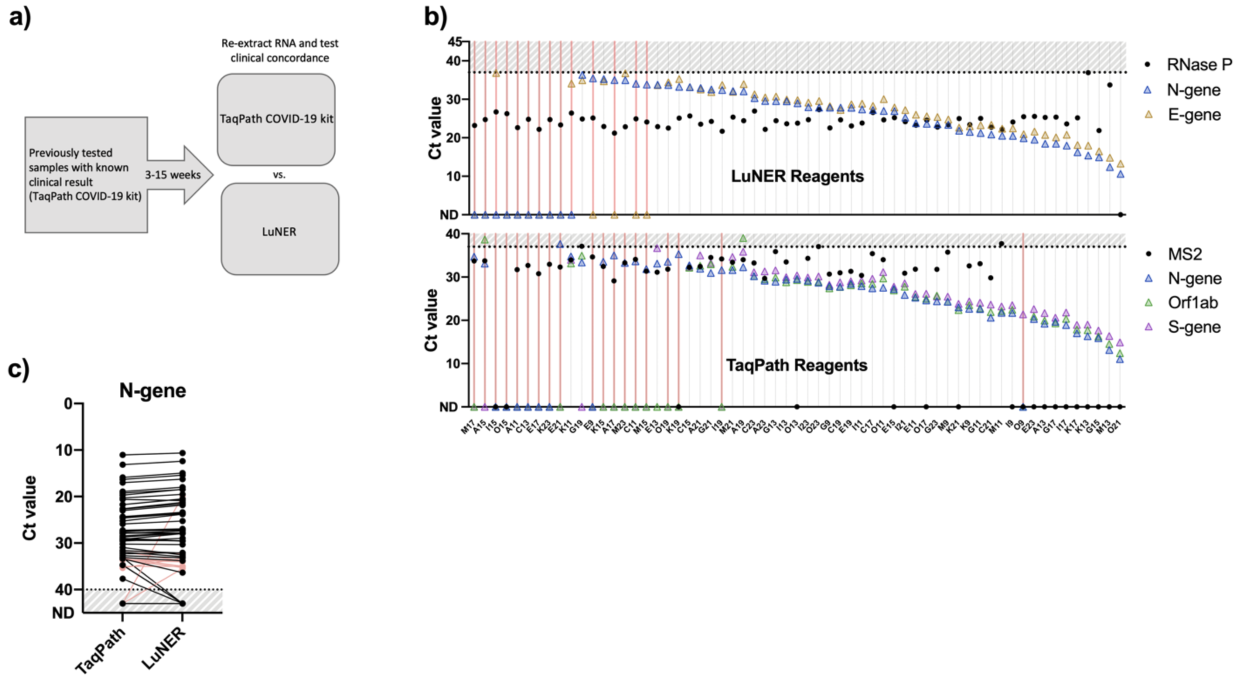

S2 Fig. Clinical concordance for samples extracted individually. a) RNA was extracted from clinical samples with previously reported results and re-analyzed with the TaqPath COVID-19 kit or LuNER assay. b) Ct value comparisons for the 61 expected positive samples. Pink lines represent sample results that differed from the original sample result. LuNER had 14 discordant results (8 negative, 6 inconclusive, 77% PPA) whereas TaqPath had 19 discordant results (6 negative, 9 inconclusive, 4 invalid, 68% PPA) out of the 61 expected positive samples. Samples with originally high Ct values were less likely to be detected upon retesting with both the TaqPath and LuNER assays, rather than samples that had been stored for the longest duration. Thus, the LuNER assay demonstrated superior sensitivity to detect positive samples in this experiment. c) Ct value comparison for N-gene between the LuNER and TaqPath assays shows strong concordance. Pink lines reflect samples in the TaqPath kit that returned inconclusive or invalid results despite amplifying N-gene with LuNER in the expected positive samples.
