# Supplementary material for: LuNER: Multiplexed SARS-CoV-2 detection in clinical swab and wastewater samples
Source: PLoS One. 2021 Nov 10;16(11):e0258263. doi: 10.1371/journal.pone.0258263 (PMC8580221; doi:10.1371/journal.pone.0258263)
Supplement: S2 File — (PDF) [file pone.0258263.s003.pdf]

LuNER: multiplexed SARS-CoV-2 detection in clinical swab and wastewater samples

Elizabeth C. Stahl[1,2], Allan R. Gopez[2], Connor A. Tsuchida[1,2], Vinson B. Fan[1], Erica A. Moehle[1,2], Lea B. Witkowsky[1,2], Jennifer R. Hamilton[1,2], Enrique Lin-Shiao[1,2], Matthew McElroy[2], Shana L. McDevitt[1,2], Alison Ciling[1,2], C. Kimberly Tsui[1], Kathleen Pestal[1], Holly K. Gildea[1], Amanda Keller[2], Iman A. Sylvain[2], Clara Williams[2], Ariana Hirsh[1,2], Alexander J. Ehrenberg[1], Rose Kantor[1], Matthew Metzger[1], IGI Testing Consortium[2^], Kara L. Nelson[1,2], Fyodor D. Urnov[1,2], Bradley R. Ringeisen[1,2], Petros Giannikopoulos[2], Jennifer A. Doudna[1,2,3*]

[1]University of California, Berkeley, Berkeley, CA, USA. [2]Innovative Genomics Institute, University of California Berkeley, Berkeley, CA, USA. [3]Howard Hughes Medical Institute, University of California, Berkeley, CA, USA.

^ Membership of the IGI Testing Consortium is provided in the acknowledgements

*Corresponding author

Email: doudna@berkeley.edu

S1 Table. Final Sample Results from Clinical Concordance of Samples Extracted Individually

	TaqPath	TaqPath	LuNER		TaqPath	TaqPath	LuNER		TaqPath	TaqPath	LuNER
Sample	*Original*	*Re-Test*	*Re-Test*	Sample	*Original*	*Re-Test*	*Re-Test*	Sample	*Original*	*Re-Test*	*Re-Test*
A1	NEG	NEG	NEG	A13	POS	POS	POS	K17	POS	POS	POS
A3	NEG	NEG	NEG	A19	POS	POS	POS	K21	POS	POS	POS
A5	NEG	NEG	NEG	A21	POS	POS	POS	M9	POS	POS	POS
A7	NEG	NEG	NEG	A23	POS	POS	POS	M11	POS	POS	POS
C1	NEG	NEG	NEG	C15	POS	POS	POS	M13	POS	POS	POS
C3	NEG	NEG	NEG	C17	POS	POS	POS	M21	POS	POS	POS
C5	NEG	NEG	NEG	C19	POS	POS	POS	O11	POS	POS	POS
C7	NEG	INVALID	NEG	C21	POS	POS	POS	O13	POS	POS	POS
E1	NEG	NEG	NEG	C23	POS	POS	POS	O17	POS	POS	POS
E3	NEG	NEG	NEG	E11	POS	POS	POS	O21	POS	POS	POS
E5	NEG	NEG	NEG	E13	POS	POS	POS	O23	POS	POS	POS
E7	NEG	NEG	NEG	E15	POS	POS	POS	K11	POS	POS	INC.
G1	NEG	NEG	NEG	E19	POS	POS	POS	A11	POS	NEG	NEG
G3	NEG	NEG	NEG	E23	POS	POS	POS	C13	POS	NEG	NEG
G5	NEG	NEG	NEG	G9	POS	POS	POS	E9	POS	NEG	INC.
I1	NEG	NEG	NEG	G11	POS	POS	POS	E17	POS	NEG	NEG
I3	NEG	NEG	NEG	G13	POS	POS	POS	E21	POS	NEG	NEG
I5	NEG	NEG	NEG	G15	POS	POS	POS	K23	POS	NEG	NEG
I7	NEG	NEG	NEG	G17	POS	POS	POS	I15	POS	INVALID	INC.
K1	NEG	NEG	NEG	G19	POS	POS	POS	K19	POS	INVALID	POS
K3	NEG	INVALID	NEG	G21	POS	POS	POS	O9	POS	INVALID	POS
K5	NEG	NEG	NEG	G23	POS	POS	POS	O15	POS	INVALID	NEG
K7	NEG	NEG	NEG	I9	POS	POS	POS	A15	POS	INC.	NEG
M1	NEG	NEG	NEG	I11	POS	POS	POS	A17	POS	INC.	INC.
M3	NEG	NEG	NEG	I13	POS	POS	POS	C11	POS	INC.	INC.
M5	NEG	INVALID	NEG	I17	POS	POS	POS	I19	POS	INC.	POS
M7	NEG	NEG	NEG	I21	POS	POS	POS	K15	POS	INC.	POS
O1	NEG	NEG	NEG	I23	POS	POS	POS	M15	POS	INC.	INC.
O3	NEG	NEG	NEG	K9	POS	POS	POS	M17	POS	INC.	NEG
O5	NEG	INVALID	NEG	K13	POS	POS	POS	M23	POS	INC.	POS
								O19	POS	INC.	POS

LuNER showed 100% negative agreement with the original sample results and 77% positive agreement with the original sample result, where the discordant samples were in 93% agreement when retested using the TaqPath RT-PCR COVID-19 kit. The TaqPath RT-PCR COVID-19 kit showed 87% negative agreement with the original sample results and 69% positive agreement with the original sample result, where the discordant samples were in 68% agreement when retested using the LuNER assay.

S2 Table. Ct Analysis of LuNER Results: Positive and Inconclusive Symptomatic (Non-pooled) Samples

	Initial Ct Values				Initial Ct Values				Initial Ct Values		
Symptomatic Samples with Final Positive Result (no retest)	RNase P	N gene	E gene	Inconclusive Primary Result, Positive Secondary Result	RNase P	N gene	E gene	Inconclusive Primary Result, Negative Secondary Result	RNase P	N gene	E gene
1	0.00	6.93	9.23	1	21.95	32.86	41.13	1	22.78	34.03	0.00
2	0.00	7.80	10.54	2	22.01	35.47	0.00	2	19.60	31.88	0.00
3	39.16	9.38	10.59	3	25.16	34.89	0.00	3	23.52	0.00	36.70
4	0.00	8.35	10.74	4	26.42	35.43	0.00	4	25.76	0.00	36.77
5	0.00	9.01	10.75	5	23.83	34.64	0.00	5	26.17	0.00	37.02
6	0.00	7.96	10.86	6	21.15	35.60	0.00	6	25.18	0.00	37.22
7	0.00	8.96	10.98	7	24.13	33.73	0.00	7	24.17	0.00	37.98
8	0.00	8.87	11.05	8	21.33	34.18	0.00	8	23.31	0.00	38.69
9	0.00	8.93	11.05	9	25.24	33.94	0.00	9	21.39	36.14	41.19
10	0.00	10.90	11.53	10	18.78	33.63	0.00	10	19.90	35.41	0.00
11	0.00	10.17	11.74	11	23.87	33.93	0.00	11	20.57	34.80	0.00
12	0.00	9.66	11.92	12	22.65	34.96	0.00	12	21.08	31.74	0.00
13	0.00	9.59	12.12	13	22.12	36.24	0.00	13	21.44	34.12	0.00
14	0.00	9.34	12.28	14	21.69	0.00	39.87	14	21.47	35.91	0.00
15	44.47	11.41	12.31	15	21.04	28.80	0.00	15	21.49	35.28	0.00
16	0.00	10.91	12.36	16	25.05	32.57	0.00	16	21.61	33.70	0.00
17	19.62	11.54	12.62	17	23.51	34.00	0.00	17	21.83	34.96	0.00
18	28.42	12.46	13.08	18	20.83	36.02	0.00	18	21.97	35.34	0.00
19	31.85	10.98	13.13	19	22.94	36.76	0.00	19	22.28	28.94	0.00
20	0.00	12.55	13.13	20	25.65	33.38	0.00	20	22.34	35.91	0.00
21	28.71	10.93	13.48	21	21.06	34.16	0.00	21	22.38	39.72	0.00
22	24.05	11.37	13.48					22	22.49	38.23	0.00
23	44.98	11.55	13.75					23	22.85	35.76	0.00
24	0.00	12.38	13.76					24	23.15	34.10	0.00
25	0.00	11.11	14.01					25	23.23	33.00	0.00
26	32.93	12.25	14.06					26	23.30	32.23	0.00
27	27.57	13.50	14.64					27	23.32	35.90	0.00
28	33.47	10.75	14.65					28	23.55	33.99	0.00

29	27.70	12.46	14.71					29	23.85	35.89	0.00
30	26.15	13.63	14.83					30	24.00	35.47	0.00
31	25.44	13.35	14.95					31	24.27	35.01	0.00
32	29.55	13.50	15.09					32	24.55	37.46	0.00
33	41.84	14.13	15.11					33	25.10	34.15	0.00
34	28.27	14.54	15.21					34	25.19	36.47	0.00
35	43.30	13.43	15.39					35	25.58	34.33	0.00
36	22.09	14.47	15.43					36	25.68	32.58	0.00
37	30.40	13.96	15.48					37	26.11	35.17	0.00
38	29.80	13.77	15.49					38	27.19	35.47	0.00
39	36.67	14.76	15.54					39	27.45	39.61	0.00
40	32.42	13.49	15.60					40	27.82	35.87	0.00
41	29.41	13.92	15.62					41	27.94	36.56	0.00
42	34.29	16.39	15.65								
43	41.26	15.00	15.81								
44	25.56	14.07	15.85								
45	32.89	15.44	15.91								
46	40.11	14.43	15.93								
47	21.39	15.15	15.94								
48	21.03	13.90	15.99								
49	27.65	14.84	16.10								
50	24.80	16.77	16.11								
51	26.00	14.92	16.20								
52	19.64	15.89	16.35								
53	21.38	15.85	16.37								
54	35.04	15.85	16.43								
55	41.63	15.73	16.46								
56	25.70	15.90	16.57								
57	31.00	16.65	16.68								
58	22.04	15.54	16.82								
59	29.56	15.29	16.98								
60	21.10	15.88	17.02								
61	22.81	15.80	17.22								
62	21.55	16.40	17.23								
63	22.88	15.64	17.32								
64	22.74	16.24	17.37								
65	19.65	15.77	17.37								
66	41.99	17.42	17.50								
67	26.53	16.39	17.59								

68	26.87	15.99	17.82								
69	28.17	15.82	17.84								
70	20.38	16.25	17.99								
71	21.26	17.33	18.12								
72	21.07	15.96	18.21								
73	20.13	16.51	18.37								
74	20.35	17.74	18.51								
75	22.91	16.17	18.51								
76	21.53	17.77	18.54								
77	21.72	17.58	18.55								
78	23.30	18.19	18.59								
79	24.74	17.53	18.84								
80	19.67	17.67	18.89								
81	22.04	17.72	18.90								
82	20.14	18.11	18.93								
83	22.25	17.76	18.96								
84	21.88	17.99	19.11								
85	23.60	18.99	19.31								
86	27.71	19.56	19.38								
87	24.66	18.91	19.51								
88	21.92	17.12	19.67								
89	22.16	17.80	19.69								
90	23.90	17.65	19.72								
91	19.97	19.85	19.95								
92	20.91	17.34	19.99								
93	24.21	17.91	19.99								
94	21.95	19.13	20.04								
95	21.95	19.38	20.13								
96	23.44	18.42	20.15								
97	22.39	18.34	20.24								
98	24.31	14.56	20.24								
99	24.39	19.28	20.29								
100	20.80	18.40	20.39								
101	22.50	19.23	20.40								
102	22.89	18.84	20.40								
103	22.70	20.18	20.60								
104	25.02	18.95	20.63								
105	21.96	20.33	20.65								
106	23.65	18.89	20.72								

107	23.81	20.43	20.73								
108	24.77	18.65	20.76								
109	21.43	19.36	20.79								
110	24.53	18.94	20.95								
111	22.69	20.11	21.02								
112	23.87	20.98	21.14								
113	21.49	20.00	21.26								
114	22.43	20.18	21.31								
115	24.37	19.77	21.36								
116	22.29	20.25	21.72								
117	25.06	20.25	21.76								
118	20.13	22.61	21.83								
119	20.87	20.41	21.89								
120	22.07	21.51	22.28								
121	21.59	22.63	22.31								
122	29.40	21.41	22.47								
123	23.92	20.82	22.52								
124	24.30	22.80	22.53								
125	22.28	20.12	22.56								
126	23.46	24.35	22.72								
127	22.15	21.90	23.19								
128	24.61	21.66	23.42								
129	23.79	21.61	23.49								
130	25.24	21.42	23.68								
131	23.71	24.29	23.79								
132	23.09	22.35	23.82								
133	22.64	22.86	24.05								
134	20.29	22.26	24.06								
135	20.51	23.90	24.13								
136	23.28	23.12	24.25								
137	22.99	24.54	24.31								
138	23.86	22.16	24.35								
139	24.04	23.39	24.49								
140	25.21	23.31	24.60								
141	20.59	23.78	24.61								
142	23.92	23.41	24.82								
143	22.21	23.85	24.83								
144	21.99	21.28	24.90								
145	21.48	24.65	24.95								

146	22.80	24.77	25.02								
147	20.16	23.37	25.05								
148	25.68	23.89	25.18								
149	25.31	25.55	25.25								
150	23.59	25.35	25.43								
151	21.68	24.90	25.88								
152	23.91	24.70	26.18								
153	20.54	24.73	26.22								
154	23.70	25.85	26.41								
155	24.31	25.58	26.47								
156	22.65	25.46	27.04								
157	20.33	24.96	27.17								
158	20.92	26.40	27.25								
159	24.86	27.13	27.28								
160	23.35	25.46	27.28								
161	20.43	26.14	27.31								
162	24.93	26.15	27.42								
163	26.06	26.93	27.51								
164	23.20	27.39	27.52								
165	22.34	26.98	27.77								
166	19.97	27.32	27.83								
167	26.32	28.29	27.90								
168	23.24	25.54	27.92								
169	25.62	25.98	28.32								
170	26.12	29.42	28.35								
171	20.49	27.51	28.41								
172	21.16	25.94	28.56								
173	24.58	27.64	28.75								
174	20.82	28.89	29.01								
175	22.47	27.28	29.20								
176	23.99	28.20	29.28								
177	22.86	25.92	29.47								
178	23.11	30.40	29.52								
179	22.56	28.66	29.55								
180	22.05	31.94	29.65								
181	25.03	31.66	29.67								
182	24.79	29.37	29.83								
183	25.67	29.92	29.86								
184	22.58	29.45	29.92								

185	21.63	29.31	29.96									
186	22.26	29.16	30.10									
187	23.18	28.60	30.45									
188	23.21	29.23	30.60									
189	24.30	29.53	30.64									
190	23.89	29.37	30.77									
191	21.58	30.38	30.93									
192	20.33	30.67	30.94									
193	20.94	29.30	31.23									
194	27.30	29.79	31.29									
195	18.56	29.30	31.37									
196	21.38	29.91	31.37									
197	22.50	29.92	31.68									
198	21.99	32.17	31.78									
199	20.52	31.11	31.94									
200	23.90	30.56	32.37									
201	20.97	31.30	32.81									
202	25.44	33.25	32.94									
203	27.33	33.80	32.98									
204	21.56	30.97	33.17									
205	22.24	31.98	33.47									
206	22.06	32.09	33.49									
207	22.11	33.85	33.49									
208	22.55	30.46	33.54									
209	21.86	31.20	33.70									
210	25.74	32.18	33.95									
211	21.92	32.94	34.02									
212	27.09	35.39	34.28									
213	25.35	34.21	34.31									
214	22.90	35.38	34.34									
215	24.23	34.57	34.40									
216	23.13	34.18	34.62									
217	20.77	31.37	34.68									
218	25.15	32.05	35.13									
219	21.66	34.97	35.34									
220	21.55	33.69	35.37									
221	21.85	35.17	35.39									
222	23.22	32.50	35.40									
223	22.98	31.01	35.74									

224	27.90	35.64	35.76								
225	26.61	35.30	35.91								
226	24.37	33.61	35.92								
227	21.32	35.44	36.60								
228	22.63	34.79	36.71								
229	22.23	33.96	36.86								
230	0.00	33.94	38.47								

After implementing the validated LuNER assay in the IGI laboratory for two weeks with a Ct cutoff of 40, we performed a post-hoc analysis of the sample results. Amplification could be occasionally observed at Ct values > 37, particularly in inconclusive samples and sample pools that would resolve as negative upon retesting. Approximately 3,018 symptomatic samples (tested individually) were resulted using the assay Ct cut-off of 40. The majority of symptomatic (89.96%) samples were negative and would not be influenced by lowering the Ct cutoff. 230 symptomatic samples were found to be positive with LuNER. Changing the Ct cutoff from 40 to 37 would change one sample result from positive to inconclusive, which would subsequently receive a retest. 62 symptomatic samples were found to be inconclusive with LuNER. 21/62 samples resolved as positive samples and lowering the Ct cutoff from 40 to 37 would result in one false negative, while 41/62 samples resolved as negative samples and lowering the Ct cutoff from 40 to 37 would reduce false inconclusive samples by 9. Red Ct values represent those whose result would be different with the change in Ct threshold.

S3 Table. Ct Analysis of LuNER Results: Positive and Inconclusive Asymptomatic (Pooled) Samples.

	Initial Ct Values				Initial Ct Values				Initial Ct Values		
Positive pools with a positive sample confirmed	RNase P	N gene	E gene	Inconclusive pools with a positive sample confirmed	RNase P	N gene	E gene	Inconclusive pools with no positive samples identified upon retest	RNase P	N gene	E gene
1	0.00	13.12	13.26	1	24.34	35.23	0.00	1	23.83	35.24	0.00
2	31.58	15.38	15.31	2	23.43	35.33	0.00	2	24.16	35.30	0.00
3	36.59	15.58	16.30	3	25.18	35.59	0.00	3	21.53	35.41	0.00
4	35.53	15.55	16.39	4	22.39	36.18	0.00	4	19.05	35.54	0.00
5	24.29	16.26	16.76	5	24.08	38.46	0.00	5	24.89	35.73	0.00
6	27.39	15.91	16.86	6	26.33	41.17	35.09	6	24.93	35.90	0.00
7	26.27	15.70	17.33	7	23.39	0.00	37.86	7	22.81	35.95	0.00
8	26.70	15.21	17.51	8	23.16	0.00	36.86	8	24.82	35.96	0.00
9	24.00	16.94	17.57					9	21.93	35.97	0.00
10	25.59	15.34	17.58					10	22.79	35.98	0.00
11	28.00	15.98	17.66					11	21.61	36.44	0.00
12	30.54	17.83	17.85					12	23.85	36.81	0.00
13	26.85	17.33	17.96					13	25.53	37.11	0.00
14	30.01	16.24	18.02					14	22.91	37.34	0.00
15	21.00	16.84	18.09					15	24.36	37.35	0.00
16	24.83	17.71	18.18					16	18.21	37.40	0.00
17	27.28	17.37	18.26					17	24.80	37.59	0.00
18	23.04	16.94	18.54					18	25.93	37.97	0.00
19	31.30	14.93	18.54					19	26.82	38.53	0.00
20	23.03	18.36	18.70					20	25.69	0.00	38.46
21	24.03	18.25	18.73					21	25.69	0.00	34.03
22	25.13	18.49	18.84					22	25.15	0.00	38.82
23	24.90	18.71	18.85					23	24.76	0.00	38.30
24	24.51	18.20	18.89					24	24.30	0.00	36.42
25	29.79	19.26	19.40					25	24.17	0.00	34.54
26	23.21	18.33	19.43					26	24.14	0.00	38.24
27	24.62	18.59	19.51					27	24.08	0.00	38.33
28	24.31	18.22	19.51					28	24.00	0.00	38.53
29	24.31	18.22	19.51					29	23.98	0.00	28.63

30	25.70	18.78	19.53					30	23.72	0.00	38.20
31	29.11	19.47	19.56					31	23.65	0.00	38.39
32	26.02	18.49	19.58					32	23.61	0.00	37.03
33	23.07	19.25	19.71					33	23.55	0.00	38.40
34	25.80	18.51	19.97					34	23.39	0.00	37.86
35	23.75	18.41	20.01					35	23.16	0.00	36.86
36	23.75	18.41	20.01					36	22.98	0.00	36.94
37	25.41	20.68	20.27					37	22.97	0.00	37.93
38	25.36	17.95	20.35					38	22.80	0.00	38.48
39	27.03	19.88	20.38					39	22.71	0.00	37.70
40	22.61	19.14	20.39					40	22.66	0.00	39.21
41	27.01	19.48	20.46					41	21.85	0.00	37.32
42	27.06	17.47	20.52								
43	25.08	19.86	20.64								
44	24.63	19.62	20.78								
45	22.31	19.70	20.80								
46	24.80	19.76	20.86								
47	25.03	20.32	20.95								
48	24.16	19.63	21.06								
49	25.22	19.90	21.10								
50	25.22	19.90	21.10								
51	22.97	19.63	21.22								
52	20.12	20.62	21.39								
53	24.35	20.38	21.46								
54	24.33	19.56	21.60								
55	24.14	21.43	21.84								
56	25.72	20.52	21.87								
57	24.40	19.40	21.88								
58	23.81	19.54	21.92								
59	23.86	19.63	21.92								
60	26.74	22.82	22.32								
61	23.28	22.73	22.56								
62	24.08	22.53	22.60								
63	22.65	22.63	22.91								
64	22.47	21.17	22.97								
65	28.35	22.53	23.47								
66	23.74	23.09	23.49								
67	23.40	21.98	23.60								
68	25.04	22.00	23.64								

69	24.47	23.50	23.67								
70	22.38	22.19	23.68								
71	24.41	23.28	23.79								
72	25.22	21.62	23.87								
73	24.12	24.25	23.88								
74	25.58	23.17	23.92								
75	23.46	23.45	23.94								
76	23.19	23.14	24.21								
77	26.19	22.76	24.26								
78	23.80	23.52	24.43								
79	22.51	23.78	24.58								
80	26.80	25.36	24.58								
81	23.48	24.84	24.89								
82	23.21	23.64	24.93								
83	24.95	22.62	24.93								
84	21.58	24.80	25.19								
85	24.03	25.58	25.35								
86	24.85	22.80	25.51								
87	26.37	24.99	25.57								
88	25.15	26.54	25.86								
89	29.09	26.14	25.93								
90	25.54	25.30	26.06								
91	23.94	23.79	26.73								
92	25.29	24.57	26.97								
93	25.55	26.15	27.71								
94	24.62	27.50	27.76								
95	24.83	27.20	28.08								
96	23.34	28.59	28.90								
97	25.60	28.40	29.16								
98	24.21	27.14	29.39								
99	25.52	28.91	29.64								
100	22.32	27.86	30.00								
101	24.12	28.89	30.02								
102	23.23	29.52	30.06								
103	22.22	30.29	30.17								
104	23.13	31.20	30.27								
105	22.60	29.27	30.64								
106	22.98	30.29	30.83								
107	25.68	29.76	30.95								

108	28.29	33.03	31.24								
109	24.30	31.72	31.25								
110	26.30	33.55	31.44								
111	23.76	30.30	31.47								
112	25.82	30.86	31.60								
113	24.26	30.90	31.69								
114	24.98	33.62	32.42								
115	24.96	32.46	32.45								
116	22.98	33.79	32.52								
117	22.91	32.80	32.93								
118	26.23	35.23	33.15								
119	26.81	31.78	33.39								
120	23.54	35.03	33.43								
121	24.22	34.34	33.92								
122	22.55	33.25	34.45								
123	24.74	34.56	34.78								
124	23.79	31.81	34.87								
125	22.50	33.31	35.26								
126	26.19	34.97	37.17								

After implementing the validated LuNER assay in the IGI laboratory for two weeks with a Ct cutoff of 40, we performed a post-hoc analysis of the sample results. Amplification could be occasionally observed at Ct values > 37, particularly in inconclusive samples and sample pools that would resolve as negative upon retesting. Approximately 28,000 asymptomatic surveillance samples (tested via fourplex pooling) were resulted using the assay Ct cut-off of 40. The majority of symptomatic (96.43%) samples were negative and would not be influenced by lowering the Ct cutoff. 126 asymptomatic pools of four samples were tested and found to be positive with LuNER. Changing the Ct cutoff from 40 to 37 would change one pool result from positive to inconclusive, which would be sent for subsequent retesting as single samples. 49 asymptomatic pools of four samples were tested and found to be inconclusive with LuNER. 8/49 of these inconclusive pools contained a positive sample, identified upon retesting of the samples

individually; lowering the Ct cutoff from 40 to 37 would result in two false negative pools, while 41/49 samples resolved into only negative samples when tested as singles, and lowering the Ct cutoff from 40 to 37 would reduce false inconclusive samples by 23 pools (92 samples). Cells marked red indicate samples for which the Ct threshold change would impact the clinical testing course.

From a combined analysis of the 1,036 symptomatic and asymptomatic samples initially found positive or inconclusive, changing the Ct cutoff from 40 to 37 would lead to 3 false negative results but would reduce retesting of false inconclusive samples by 100 tests. In a testing lab that performs thousands of tests per day, a streamlined testing pipeline is critical; the process of re-identifying individual samples within these thousands of samples and re-testing them disrupts the routine and leads to increased turnaround times. Therefore, we reasoned that the benefits of lowering the Ct cutoff from 40 to 37 outweighed the increase in potential false negative results. This could be reevaluated when sample throughput is lower, or turnaround times are less critical.

S4 Table. Transition of duplex qPCR reactions with RNase P and N1 from Biorad CFx96 to QuantStudio-6 (QS6) 384-well plates with 2x and 4x NEB master mix (Fig 1 raw data).

PCR Machine & Master mix	Reaction Size	Plasmid	Replicate	Ct values<br>N1 probe FAM	Ct values<br>RNase P probe ATTO 647
CFx96 2x	Full Reaction	N1 only	1	31.06	0.00
			2	30.62	0.00
			3	30.70	0.00
		RNase P only	1	0.00	30.88
			2	0.00	30.77
			3	0.00	30.88
		N1 and RNase P	1	30.48	30.93
			2	30.12	30.84
			3	30.23	30.71
		No plasmid	1	0.00	0.00
			2	0.00	0.00
			3	0.00	0.00
	Half Reaction	N1 only	1	31.05	0.00
			2	31.15	0.00
			3	31.05	0.00
		RNase P only	1	0.00	31.36
			2	0.00	32.26
			3	0.00	31.73
		N1 and RNase P	1	31.70	31.88
			2	31.14	31.52
			3	31.11	31.47
		No plasmid	1	0.00	0.00
			2	0.00	0.00
			3	0.00	0.00
QS6 2x	Full Reaction	N1 only	1	28.70	0.00
			2	28.89	0.00
			3	28.64	0.00
		RNase P only	1	0.00	29.89
			2	0.00	30.32
			3	0.00	30.67
		N1 and RNase P	1	28.25	29.60
			2	28.90	30.26
			3	28.73	30.28

		No plasmid	1	0.00	0.00
			2	0.00	0.00
			3	0.00	0.00
	Half Reaction	N1 only	1	28.45	0.00
			2	28.55	0.00
			3	28.59	0.00
		RNase P only	1	0.00	30.99
			2	0.00	30.87
			3	0.00	31.04
		N1 and RNase P	1	29.34	30.94
			2	29.34	31.32
			3	29.24	31.53
		No plasmid	1	0.00	0.00
			2	0.00	0.00
			3	0.00	0.00
QS6 4x	Full Reaction	N1 only	1	28.00	0.00
			2	28.36	0.00
			3	28.38	0.00
		RNase P only	1	0.00	30.19
			2	0.00	30.12
			3	0.00	30.38
		N1 and RNase P	1	28.52	29.93
			2	28.85	29.94
			3	29.13	30.11
		No plasmid	1	0.00	0.00
			2	0.00	0.00
			3	0.00	0.00
	Half Reaction	N1 only	1	28.11	0.00
			2	28.46	0.00
			3	28.34	0.00
		RNase P only	1	0.00	31.28
			2	0.00	31.09
			3	0.00	31.01
		N1 and RNase P	1	29.41	30.78
			2	28.92	30.93
			3	29.36	31.88
		No plasmid	1	0.00	0.00
			2	0.00	0.00
			3	0.00	0.00

qPCR was performed on positive control plasmids for N1 and RNase P on the Bio-Rad CFx96. The duplexed reactions were successfully adapted to the Applied Biosystems QuantStudio-6 using half reaction volumes and 384-well plate format after custom dye calibration to detect ATTO 647 with both the standard 2x and a newly developed 4x Luna Probe One-Step RT-qPCR master mix from New England BioLabs. Plasmid DNA was added directly into the master mix at 200,000 copies/mL (2-4x Saliva Direct limit of detection) in triplicate. Full reactions (15µL master mix with 5µL sample input), half reactions (7.5µL master mix with 5µL sample input).

S5 Table. Duplex qPCR reactions with RNase P and E-Sarbeco or RdRp on QuantStudio-6 (QS6) 384-well plates with 4x NEB master mix formulations (Fig 2 a,b raw data).

PCR Machine & Master mix	Reaction Size	Plasmid	Replicate	Ct values: E-gene probe FAM	Ct values: RNase P probe ATTO 647
QS6 4x	Full Reaction	E-Sarbeco only	1	26.99	0.00
			2	28.93	0.00
			3	29.42	0.00
		RNase P only	1	0.00	30.20
			2	0.00	29.83
			3	0.00	30.34
		E and RNase P	1	28.99	31.31
			2	28.95	31.34
			3	28.73	31.04
		No plasmid	1	0.00	0.00
			2	0.00	0.00
			3	0.00	0.00
	Half Reaction	E-Sarbeco only	1	30.54	0.00
			2	29.99	0.00
			3	30.53	0.00
		RNase P only	1	0.00	29.99
			2	0.00	30.48
			3	0.00	30.41
		E and RNase P	1	28.29	30.97
			2	28.89	30.61
			3	28.80	31.52
		No plasmid	1	0.00	0.00
			2	0.00	0.00
			3	0.00	0.00
				**RdRp probe FAM**	**RNase P probe ATTO 647**
QS6 4x	Full Reaction	RdRp only	1	27.77	0.00
			2	36.29	0.00
			3	27.66	0.00
		RNase P only	1	0.00	30.66
			2	0.00	30.28
			3	0.00	29.84
			1	30.51	31.11

		RdRp and RNase P	2	30.64	31.82
			3	30.16	31.10
		No plasmid	1	0.00	0.00
			2	0.00	0.00
			3	0.00	0.00
	Half Reaction	RdRp only	1	30.22	0.00
			2	30.66	0.00
			3	31.38	0.00
		RNase P only	1	0.00	30.93
			2	0.00	30.40
			3	0.00	30.53
		RdRp and RNase P	1	31.64	30.80
			2	31.34	30.33
			3	31.64	30.41
		No plasmid	1	0.00	0.00
			2	0.00	0.00
			3	0.00	0.00

Duplex qPCR was performed on positive control plasmids for E-Sarbeco and RdRp on the QuantStudio-6 using 384-well plate with 4x Luna Probe One-Step RT-qPCR master mix to evaluate the use of either primer/probe set as the third component of the multiplexed assay. Plasmid DNA was added directly into the master mix at 200,000 copies/mL (2-4x Saliva Direct limit of detection) in triplicate.

S6 Table. Effect of concentration and mismatch in RdRp reverse primer on qPCR RdRp detection (Fig 2c raw data).

RdRp Reverse Primer	Concentration (nM)	Replicate	Ct Value
Mismatch	400	1	31.67
		2	31.28
		3	31.43
	600	1	26.30
		2	27.01
		3	27.23
Corrected	400	1	30.24
		2	30.43
		3	30.36
	600	1	26.89
		2	27.20
		3	26.97

The RdRp reverse primer is recommended to be used at 600 nM (compared to 400nM for N1 and E-Sarbeco) and contains a mismatch at position 12 where S should be corrected to T to match the SARS-CoV-2 genome. At 400nM, correcting the mismatch improved the average Ct value by 1.1. At 600nM, there was no difference in Ct values between the mismatch and corrected primers.

S7 Table. Multiplex qPCR reactions with RNase P, N1, and E-Sarbeco on QuantStudio-6 (QS6) 384-well plates with 4x NEB master mix formulation (Fig 2d raw data).

PCR Machine & Master mix	Reaction Size	Plasmid	Replicate	E-gene probe SUN	RNase P probe ATTO 647	N-gene probe FAM
QS6 4x	Half reaction	E-Sarbeco only	1	27.40	0.00	0.00
			2	26.96	0.00	0.00
			3	27.32	0.00	0.00
		N1 only	1	0.00	0.00	26.25
			2	0.00	0.00	26.27
			3	0.00	0.00	26.35
		E-Sarbeco and RNase P	1	27.20	29.65	0.00
			2	27.78	27.23	0.00
			3	28.56	27.15	0.00
		E-Sarbeco, N1, and RNase P	1	28.71	28.36	26.78
			2	29.03	29.07	27.25
			3	28.40	28.62	26.99
		No plasmid	1	0.00	0.00	0.00
			2	0.00	0.00	0.00
			3	0.00	0.00	0.00

The E-Sarbeco primers and probes were successfully multiplexed in qPCR reactions using the 4x master mix formulations at final primer concentration of 400nM, demonstrating superior performance over RdRp. Therefore, we have selected the 4x Luna master mix to detect N-gene, E-gene, and RNase P (NER) as the final formulation of our multiplexed assay, hereafter referred to as LuNER.

S8 Table. Limit of detection (LoD) of LuNER assay with fourplex pooled samples containing heat-inactivated SARS-CoV-2 (Fig 3a,b raw data).

Sample ($TCID_{50}$/mL)	Replicate	Ct values<br>RNase P probe ATTO 647	E-gene probe SUN	N-gene probe FAM
10.24	1	24.06	32.63	30.87
	2	24.04	32.25	30.61
	3	23.91	32.41	30.74
5.26	1	23.89	34.01	31.53
	2	24.10	33.73	31.26
	3	23.94	33.45	31.18
2.56	1	23.83	34.68	32.96
	2	24.13	35.29	32.20
	3	24.00	34.94	32.19
1.28	1	23.91	36.48	34.60
	2	24.10	35.45	32.23
	3	24.15	36.85	0.00
0.64	1	24.08	36.83	35.33
	2	24.13	35.14	34.12
	3	24.10	36.48	33.53
0.32	1	24.31	37.77	34.17
	2	24.15	0.00	0.00
	3	24.07	36.84	0.00
0.16	1	24.18	0.00	0.00
	2	23.97	0.00	34.33
	3	23.99	0.00	34.57
0.08	1	24.11	0.00	44.97
	2	24.06	0.00	0.00
	3	24.03	0.00	0.00
0.04	1	23.85	0.00	0.00
	2	23.93	0.00	0.00
	3	23.83	0.00	0.00
Human RNA Control	1	23.88	0.00	0.00
	2	23.96	0.00	0.00
	3	23.87	0.00	0.00
Negative RNA Control	1	0.00	0.00	0.00
	2	0.00	0.00	0.00
	3	0.00	0.00	0.00
	1	0.00	0.00	0.00

RT-qPCR Negative Control	2	0.00	0.00	0.00
	3	0.00	0.00	0.00
	4	0.00	0.00	0.00
RT-qPCR Positive Control	1	0.00	26.21	26.57
	2	0.00	27.16	27.28
	3	0.00	26.42	26.92
	4	0.00	26.80	27.10

Heat-inactivated SARS-CoV-2 was introduced into negative matrix generated from one clinically reported negative sample and combined with equal volumes of three additional negative samples to mimic the fourplex pooling protocol, prior to RNA extraction and qRT-PCR. RNase P was detected in all samples, while either N-gene or E-gene were detectable in samples at increasing Ct-values from 10.24 to 0.64 $TCID_{50}$/mL. All controls were valid.

S9 Table. Reproducibility of limit of detection (LoD) of LuNER assay with fourplex pooled samples containing heat-inactivated SARS-CoV-2 (Fig 3c raw data).

		Ct values		
**Sample (TCID50/mL)**	**Replicate**	**RNase P probe ATTO 647**	**E-gene probe SUN**	**N-gene probe FAM**
2.56	1	24.87	34.64	32.04
	2	24.65	34.04	30.94
	3	24.61	0.00	31.28
	4	24.73	34.56	31.39
	5	24.78	0.00	33.62
	6	24.89	0.00	32.38
	7	24.84	0.00	31.47
	8	24.71	34.86	31.34
	9	24.64	44.38	31.71
	10	24.76	0.00	32.39
	11	24.62	34.76	31.14
	12	24.81	0.00	31.10
	13	24.86	0.00	34.10
	14	24.84	34.64	31.27
	15	24.67	0.00	30.83
	16	24.88	0.00	33.98
	17	24.59	0.00	30.92
	18	24.58	35.32	31.84
	19	24.59	34.63	31.52
	20	24.76	35.47	31.70
1.28	1	24.64	0.00	32.86
	2	24.71	0.00	32.85
	3	24.72	0.00	31.72
	4	24.51	35.30	32.40
	5	23.96	35.08	33.95
	6	24.57	0.00	34.89
	7	24.48	0.00	32.35
	8	24.47	0.00	33.14
	9	24.75	0.00	32.61
	10	24.42	0.00	31.68
	11	24.56	0.00	32.56
	12	24.23	34.56	31.40

	13	24.53	0.00	33.02
	14	24.48	0.00	33.08
	15	24.50	0.00	32.86
	16	24.66	0.00	33.00
	17	24.65	36.22	32.13
	18	24.62	0.00	31.83
	19	24.51	34.81	32.25
	20	24.32	0.00	32.32
0.64	1	24.62	0.00	33.90
	2	24.43	0.00	31.78
	3	24.43	0.00	0.00
	4	24.08	0.00	32.93
	5	24.32	0.00	34.36
	6	24.60	36.18	33.25
	7	24.51	0.00	34.84
	8	24.41	0.00	33.91
	9	24.51	0.00	32.76
	10	24.53	0.00	32.34
	11	24.39	36.96	35.05
	12	24.14	0.00	33.21
	13	24.36	0.00	31.93
	14	24.26	0.00	32.66
	15	24.41	0.00	32.42
	16	24.63	0.00	37.56
	17	24.51	0.00	33.82
	18	24.47	37.45	34.25
	19	24.58	0.00	32.25
	20	23.74	0.00	32.83

Heat-inactivated SARS-CoV-2 was introduced into negative matrix generated from one clinically reported negative sample and combined with equal volumes of three additional negative samples to mimic the fourplex pooling protocol, prior to RNA extraction and qRT-PCR. RNase P was detected in all samples, while either N-gene or E-gene were detectable in 20/20 at 1.28 and 2.56 TCID50/mL and 19/20 at 0.64 TCID50/mL.

S10 Table. Clinical Concordance of LuNER Assay in Expected Positive Sample Pools (Fig 4a raw data).

	Ct Values from Pooled Samples		
**Expected Positive Pools**	**Rnase P**	**E-gene**	**N-gene**
1	20.84	19.12	16.01
2	38.23	16.88	14.76
3	22.90	28.65	26.60
4	27.59	18.81	17.37
5	23.90	20.39	19.45
6	22.91	24.43	22.99
7	22.84	25.43	23.86
8	33.28	15.80	13.15
9	22.49	17.77	17.29
10	26.03	16.97	15.71
11	31.90	19.52	17.90
12	22.24	29.71	27.11
13	23.22	20.75	19.84
14	25.25	20.42	18.64
15	42.87	16.78	15.44
16	0.00	15.47	14.28
17	25.77	18.01	16.76
18	28.62	17.65	15.90
19	21.95	19.82	19.27
20	31.48	18.29	16.84
21	23.61	21.06	18.66
22	22.53	20.71	17.90
23	24.37	29.97	28.23
24	23.65	27.10	25.79
25	24.45	25.74	24.61
26	23.76	24.62	22.34
27	0.00	12.96	10.71
28	25.23	21.85	18.27
29	0.00	16.04	14.43
30	21.59	21.85	21.21

Thirty samples that were previously reported positive for SARS-CoV-2 using the ThermoFisher TaqPath RT-qPCR assay were arrayed into deep well plates, along with three negative samples,

to create sample pools of four. The pools underwent RNA extraction and analysis with the LuNER assay. All thirty pools amplified N-gene or E-gene, triggering resting of all 120 samples individually (100% PPA).

S11 Table. Clinical Concordance of LuNER Assay in Expected Negative Sample Pools (Fig 4b raw data).

	Ct Values from Pooled Samples		
**Expected Negative Pools**	**Rnase P**	**E-gene**	**N-gene**
1	23.92	0.00	0.00
2	25.14	0.00	0.00
3	25.28	0.00	0.00
4	20.87	0.00	0.00
5	23.61	0.00	0.00
6	23.50	0.00	36.24
7	23.75	0.00	0.00
8	25.26	0.00	0.00
9	24.51	0.00	0.00
10	24.39	0.00	0.00
11	22.64	0.00	0.00
12	23.89	0.00	0.00
13	23.83	0.00	0.00
14	25.98	0.00	0.00
15	24.64	0.00	0.00
16	25.16	0.00	0.00
17	25.13	0.00	0.00
18	24.87	0.00	0.00
19	25.23	0.00	0.00
20	21.97	0.00	0.00
21	24.45	0.00	0.00
22	25.51	0.00	0.00
23	24.91	0.00	0.00
24	25.90	0.00	0.00
25	24.91	0.00	0.00
26	24.01	0.00	0.00
27	24.70	0.00	0.00
28	23.89	0.00	0.00
29	23.57	0.00	0.00
30	25.16	0.00	0.00

Thirty samples that were previously reported negative for SARS-CoV-2 using the ThermoFisher TaqPath RT-qPCR assay were arrayed into deep well plates, along with three additional negative

samples, to create sample pools of four. The pools underwent RNA extraction and analysis with the LuNER assay. One of the thirty pools (pool 6, red text) amplified N-gene below the Ct cut-off of 37, thus these four samples were sent for individual retesting. The remaining 116 samples were resulted as negative (96.6% PNA).

S12 Table. Clinical Concordance of LuNER Assay – Individual Testing (Fig 4c raw data).

		Ct Values from Individual Samples		
**Pool**	**Sample**	**RNase P**	**E-gene**	**N-gene**
1	1	17.78	17.82	12.76
	2	24.12	0.00	0.00
	3	23.91	0.00	0.00
	4	25.12	0.00	0.00
2	5	0.00	14.96	12.53
	6	24.15	0.00	0.00
	7	23.14	0.00	0.00
	8	23.47	0.00	0.00
3	9	20.66	26.09	24.22
	10	26.67	0.00	0.00
	11	24.59	0.00	0.00
	12	23.88	0.00	0.00
4	13	0.00	17.43	15.24
	14	24.79	0.00	0.00
	15	23.62	0.00	0.00
	16	24.44	0.00	36.92
5	17	24.45	19.11	17.01
	18	29.67	0.00	0.00
	19	25.58	0.00	0.00
	20	21.45	0.00	0.00
6	21	21.06	22.32	21.03
	22	27.16	0.00	0.00
	23	23.83	0.00	0.00
	24	22.77	0.00	0.00
7	25	20.71	23.29	21.65
	26	25.27	0.00	0.00
	27	28.17	0.00	0.00
	28	24.73	0.00	40.93
8	29	0.00	14.22	11.21
	30	25.70	0.00	0.00
	31	27.35	0.00	0.00
	32	20.79	0.00	0.00
9	33	21.95	18.90	16.78
	34	26.79	0.00	0.00
	35	24.79	0.00	0.00

	36	27.34	0.00	0.00
10	37	27.80	14.48	13.12
	38	22.05	0.00	0.00
	39	24.49	0.00	0.00
	40	25.88	0.00	0.00
11	41	41.64	17.43	15.79
	42	26.83	0.00	0.00
	43	26.56	0.00	0.00
	44	25.27	0.00	0.00
12	45	21.72	27.40	25.35
	46	20.59	0.00	0.00
	47	23.36	0.00	0.00
	48	25.62	0.00	0.00
13	49	21.52	18.97	17.72
	50	24.82	0.00	0.00
	51	23.51	0.00	0.00
	52	22.97	0.00	0.00
14	53	24.23	18.78	16.48
	54	24.69	0.00	0.00
	55	25.79	0.00	0.00
	56	24.50	0.00	0.00
15	57	0.00	15.65	13.75
	58	23.76	0.00	0.00
	59	25.54	0.00	0.00
	60	26.87	0.00	0.00
16	61	0.00	12.86	12.12
	62	25.22	0.00	0.00
	63	23.19	0.00	0.00
	64	24.58	0.00	0.00
17	65	29.56	16.20	14.61
	66	28.77	0.00	0.00
	67	24.79	0.00	0.00
	68	23.50	0.00	0.00
18	69	41.38	16.08	13.61
	70	23.66	0.00	0.00
	71	25.79	0.00	0.00
	72	21.95	0.00	0.00
19	73	21.33	17.91	17.16
	74	24.63	0.00	33.97

	75	22.44	0.00	0.00
	76	25.14	0.00	0.00
20	77	0.00	15.91	14.34
	78	25.44	0.00	0.00
	79	24.29	0.00	0.00
	80	24.58	0.00	0.00
21	81	22.93	18.89	16.45
	82	26.11	0.00	0.00
	83	20.88	0.00	33.70
	84	27.39	0.00	0.00
22	85	23.77	18.04	15.97
	86	24.44	0.00	0.00
	87	27.06	0.00	0.00
	88	24.49	0.00	0.00
23	89	22.81	28.15	26.69
	90	24.80	0.00	0.00
	91	26.22	0.00	0.00
	92	26.79	0.00	0.00
24	93	22.72	24.70	23.44
	94	25.86	0.00	40.06
	95	24.13	0.00	0.00
	96	23.12	0.00	0.00
25	97	23.22	23.89	22.54
	98	24.61	0.00	0.00
	99	24.92	0.00	0.00
	100	23.90	0.00	0.00
26	101	22.25	22.87	20.41
	102	26.28	0.00	0.00
	103	27.99	0.00	0.00
	104	23.18	0.00	0.00
27	105	0.00	0.00	8.54
	106	25.44	0.00	0.00
	107	25.51	0.00	0.00
	108	24.30	0.00	0.00
28	109	33.72	19.95	15.71
	110	22.71	0.00	0.00
	111	25.52	0.00	0.00
	112	26.40	0.00	0.00
29	113	0.00	14.66	12.66

	114	25.39	0.00	0.00
	115	26.48	0.00	0.00
	116	26.67	0.00	0.00
30	117	20.45	30.70	25.12
	118	22.99	20.15	18.62
	119	24.13	0.00	0.00
	120	25.90	0.00	0.00
31 (expected negative pool 6)	121	26.95	35.38	34.13
	122	26.29	0.00	0.00
	123	26.74	0.00	0.00
	124	22.20	0.00	0.00

Thirty-one pools (124 samples) were tested individually based on the measured amplification of N-gene or E-gene when tested as a pool of four. The samples underwent RNA extraction and analysis with the LuNER assay. Each pool is expected to contain a single positive sample; however, four pools were found to contain an additional positive or inconclusive sample and one pool contained one inconclusive sample only (red text, PPA = 84%). The additional positive sample identified in pool 30 (sample 118) was determined to be a true positive based on the original result with TaqPath qRT-PCR and was included in this experiment by error. The remaining four inconclusive samples were sent for retesting.

S13 Table. Clinical Concordance of LuNER Assay – Inconclusive Retests (Fig 4d raw data).

		Ct Values from Individual Samples		
**Pool**	**Sample**	**RNaseP**	**E-gene**	**N-gene**
4	16	24.53	0.00	0.00
19	74	24.79	0.00	0.00
21	83	22.39	0.00	0.00
27	105	0.00	0.00	8.68

Four inconclusive samples underwent RNA extraction and analysis with the LuNER assay. Three of the four were found to be negative upon retesting, therefore the final sample result was negative. This agrees with the original sample result obtained with TaqPath RT-qPCR (100% PNA). One inconclusive sample again showed amplification of N-gene only at a low Ct value, therefore the final sample result was positive. This agrees with the original result obtained with TaqPath RT-qPCR (100% PPA).

S14 Table. PCR efficiency of N1 and E-Sarbeco on QuantStudio-3 (Fig 5a raw data).

		Ct Values	
**Copies per reaction**	**Replicate**	**N-gene**	**E-gene**
2.5	1	33.82	34.02
	2	33.08	36.78
	3	35.34	37.35
5	1	34.35	34.60
	2	33.13	34.09
	3	34.16	34.39
10	1	32.58	33.16
	2	33.37	32.79
	3	32.33	32.28
50	1	29.97	30.27
	2	30.06	30.16
	3	29.97	30.11
500	1	26.69	27.34
	2	26.51	27.01
	3	26.62	27.20
5000	1	24.53	24.67
	2	24.00	24.58
	3	23.34	23.44
50000	1	20.58	21.12
	2	20.87	21.40
	3	20.62	21.34

Duplex qPCR was performed on positive control plasmids for E-Sarbeco and N1 on the QuantStudio-3 using 96-well plates with 4x Luna Probe One-Step RT-qPCR master mix to adapt to the COVID wastewater epidemiology for the Bay Area (COVID-WEB) pop-up testing laboratory. Plasmid DNA was added directly into the master mix in triplicate and plotted to determine PCR efficiency.

S15 Table. Surveillance of SARS-CoV-2 in wastewater with LuNER assay (Fig 5b raw data).

		**Ct Values**		
Site	Replicate	RNase P	E-gene	N-gene
1	1	32.45	0.00	36.16
	2	37.68	0.00	0.00
	3	33.49	0.00	36.28
2	1	30.85	35.68	33.25
	2	31.62	34.83	33.73
	3	32.15	33.74	33.75
3	1	0.00	33.94	34.21
	2	35.89	35.12	32.45
	3	34.11	34.88	32.66
4	1	32.54	33.91	32.00
	2	33.98	35.02	31.71
	3	32.74	34.63	32.37
5	1	32.31	31.77	30.21
	2	32.74	32.44	30.51
	3	32.53	31.94	30.45
6	1	31.53	32.24	30.25
	2	32.47	32.26	30.65
	3	32.29	33.00	30.43
7	1	29.78	31.99	30.11
	2	30.80	31.97	30.03
	3	30.87	31.77	29.85
8	1	31.31	35.47	32.86
	2	31.56	35.06	32.95
	3	32.12	34.10	32.87
9	1	33.71	32.63	30.73
	2	39.65	32.49	30.86
	3	34.53	33.59	31.42
Negative Control	1	0.00	0.00	0.00
	2	0.00	0.00	0.00
	3	0.00	0.00	0.00

Wastewater collected from nine different sites was found to contain viral RNA with LuNER, as expected. However, site 1 has an overall inconclusive result and would be retested.

S16 Table. N-gene concordance in wastewater with LuNER (Fig 5c raw data).

	N-gene Ct Values		
Site	Replicate	Current Test	LuNER
1	1	0.00	36.16
	2	37.99	0.00
	3	35.69	36.28
2	1	35.18	33.25
	2	34.85	33.73
	3	35.48	33.75
3	1	36.32	34.21
	2	34.38	32.45
	3	34.17	32.66
4	1	34.69	32.00
	2	35.08	31.71
	3	34.73	32.37
5	1	32.50	30.21
	2	32.77	30.51
	3	32.67	30.45
6	1	33.00	30.25
	2	33.23	30.65
	3	32.51	30.43
7	1	32.03	30.11
	2	31.91	30.03
	3	32.03	29.85
8	1	34.95	32.86
	2	35.51	32.95
	3	34.77	32.87
9	1	33.33	30.73
	2	33.88	30.86
	3	33.25	31.42
Negative Control	1	0.00	0.00
	2	0.00	0.00
	3	0.00	0.00

Similar Ct values for N-gene values obtained between the current test used for wastewater testing and LuNER.

S17 Table. Limit of detection (LoD) of LuNER assay with individual samples containing heat-inactivated SARS-CoV-2 (S1 a,c raw data).

		**Ct Values**		
**Sample (TCID50/mL)**	**Replicate**	**RNase P probe ATTO 647**	**N-gene probe FAM**	**E-gene probe SUN**
50	1	24.43	26.50	28.42
	2	24.34	26.03	28.47
	3	24.28	26.19	28.57
10	1	24.43	29.37	30.82
	2	24.48	29.38	30.95
	3	24.46	28.96	30.96
5	1	24.60	31.66	32.95
	2	24.40	30.69	32.19
	3	24.27	31.30	32.10
1	1	24.65	33.84	36.60
	2	24.56	34.26	34.15
	3	24.55	34.81	34.08
0.5	1	24.58	33.96	34.49
	2	24.46	34.53	36.49
	3	24.61	33.63	40.31
0.1	1	24.50	0.00	36.72
	2	24.55	0.00	0.00
	3	24.44	0.00	34.61
RT-qPCR Negative Control	1	0.00	42.66	0.00
	2	0.00	0.00	0.00
	3	0.00	0.00	0.00
	4	0.00	0.00	0.00
RT-qPCR Positive Control	1	28.73	30.34	28.19
	2	28.88	29.86	28.52
	3	28.53	30.34	28.23
	4	28.88	29.94	28.01
Human RNA Control	1	23.47	0.00	0.00
Negative RNA Control	1	0.00	0.00	0.00

Heat-inactivated SARS-CoV-2 was introduced into negative matrix generated from one clinically reported negative sample, prior to RNA extraction and qRT-PCR. RNase P was detected in all samples, while either N-gene or E-gene were detectable in samples at increasing Ct-values from 50 to 0.1 $TCID_{50}$/mL. All controls were valid.

S18 Table. Reproducibility with LuNER assay and individual samples containing heat-inactivated SARS-CoV-2 (S1 b raw data).

	Ct values		
**Replicate**	**RNase P probe ATTO 647**	**E-gene probe SUN**	**N-gene probe FAM**
1	20.60	31.82	31.70
2	20.26	31.44	30.25
3	20.44	31.50	30.19
4	20.35	30.90	30.12
5	20.31	31.60	30.30
6	20.26	31.77	30.62
7	20.44	31.13	31.16
8	20.55	31.54	30.87
9	20.48	32.69	32.51
10	20.38	31.31	29.35
11	20.30	30.90	30.18
12	20.32	31.33	30.48
13	20.34	31.17	29.95
14	20.49	31.53	31.08
15	20.37	31.79	30.48
16	20.35	31.44	30.84
17	20.40	31.90	31.58
18	20.36	31.63	30.54
19	20.24	31.41	30.26
20	20.24	31.42	29.65

Twenty replicates at 1 $TCID_{50}$/mL were prepared by extracting RNA from heat inactivated virus and performing RT-qPCR with the LuNER reagents to test for reproducibility at 2x individual sample LoD.

S19 Table. Reproducibility with LuNER assay and individual samples containing heat-inactivated SARS-CoV-2 (S2 b,c and S1 Table raw data).

	LuNER (Ct Values)			TaqPath (Ct Values)			
Sample Well	RNase P	N-gene	E-gene	MS2	N-gene	Orf1ab	S-gene
M17	23.19	0.00	0.00	33.72	34.72	0.00	0.00
A15	24.73	0.00	0.00	33.75	33.13	38.68	0.00
I15	26.73	0.00	36.84	0.00	0.00	0.00	0.00
O15	26.27	0.00	0.00	0.00	0.00	0.00	0.00
A11	22.64	0.00	0.00	31.73	0.00	0.00	0.00
C13	24.82	0.00	0.00	32.69	0.00	0.00	0.00
E17	22.18	0.00	0.00	30.77	0.00	0.00	0.00
K23	24.74	0.00	0.00	32.96	0.00	0.00	0.00
E21	23.36	0.00	0.00	32.31	37.66	0.00	0.00
K11	26.40	0.00	34.11	33.95	34.75	33.16	33.98
G19	24.88	36.37	34.96	37.13	33.39	34.96	0.00
E9	25.13	35.47	0.00	34.65	0.00	0.00	0.00
K15	22.93	35.27	34.70	32.45	33.56	0.00	0.00
A17	21.21	35.02	0.00	29.12	35.01	0.00	0.00
M23	22.86	34.96	36.78	33.27	33.27	0.00	0.00
C11	24.91	34.05	0.00	34.07	33.66	0.00	0.00
M15	24.11	33.83	0.00	31.36	31.85	0.00	0.00
E13	22.90	33.82	33.71	31.13	33.11	0.00	36.71
O19	22.53	33.73	34.39	31.81	33.54	0.00	0.00
K19	25.12	33.26	35.27	0.00	35.33	0.00	0.00
C15	25.65	33.16	33.15	32.26	32.72	32.19	32.34
A21	23.54	32.94	32.57	32.46	31.94	32.60	35.02
G21	24.25	32.58	31.91	34.53	30.94	32.94	33.25
I19	21.69	32.40	33.73	34.19	31.60	0.00	0.00
M21	25.38	32.17	31.96	33.42	31.61	32.38	34.65
A19	24.43	32.07	33.97	34.00	32.30	39.00	35.86
C23	26.96	30.31	31.29	33.24	30.20	30.22	31.10
A23	22.18	29.56	30.49	29.67	29.18	29.51	31.33
G13	24.43	29.54	30.74	35.89	28.96	29.87	31.55
I13	23.65	29.51	29.95	33.47	29.42	28.81	29.99
O13	23.79	28.96	29.79	0.00	29.58	29.30	30.38
I23	24.71	27.97	29.14	34.33	29.19	28.90	29.90
O23	27.31	27.85	29.57	37.05	28.70	28.93	30.14

G9	22.56	27.85	28.22	30.67	27.90	27.43	28.21
C19	24.66	27.84	27.23	30.97	27.78	27.72	28.77
E19	23.09	27.82	28.69	31.33	28.46	28.10	29.00
I11	23.83	27.41	28.88	30.37	27.91	28.54	29.21
C17	26.58	27.19	28.31	35.41	27.41	28.43	29.61
O11	24.67	26.97	30.06	34.01	27.56	29.69	31.16
E15	25.22	26.96	27.88	0.00	27.24	26.91	27.79
I21	24.16	25.26	27.20	30.87	25.89	27.77	28.54
E11	23.26	23.78	25.99	31.82	25.24	25.40	26.13
O17	24.59	23.63	25.61	0.00	24.62	24.99	26.14
G23	22.79	23.52	25.40	31.77	24.40	25.71	25.53
M9	23.39	23.37	24.76	35.74	24.31	24.35	25.49
K21	25.01	21.83	22.64	0.00	23.01	22.39	23.80
K9	23.40	21.54	23.01	32.57	22.69	23.50	24.39
G11	25.03	21.25	23.25	33.07	22.63	22.79	24.07
C21	22.73	20.87	23.37	29.83	20.62	21.91	23.65
M11	22.23	20.54	22.38	37.72	21.75	22.16	23.22
I9	24.10	20.50	22.45	0.00	21.73	22.46	23.51
O9	25.48	19.90	20.93	0.00	0.00	0.00	21.38
E23	25.56	19.51	21.62	0.00	20.30	20.88	22.60
A13	25.30	18.47	20.74	0.00	19.26	19.94	21.67
G17	25.36	18.45	20.13	0.00	19.69	19.33	20.63
I17	23.62	17.98	20.78	0.00	18.92	20.34	21.81
K17	25.18	16.26	18.12	0.00	17.00	17.87	18.95
K13	36.93	15.38	17.99	0.00	16.34	17.75	19.01
G15	21.89	14.96	16.49	0.00	15.88	16.28	17.64
M13	33.73	12.38	14.84	0.00	13.14	14.54	16.39
O21	0.00	10.64	13.31	0.00	11.04	12.42	14.91

Ct value comparisons for the 61 expected positive samples. LuNER had 14 discordant results (8 negative, 6 inconclusive, 77% PPA) whereas TaqPath had 19 discordant results (6 negative, 9 inconclusive, 4 invalid, 68% PPA), when compared to the original TaqPath sample result.
